# Nonlinear PHQ-9 thresholds and mortality in cardiovascular-kidney-metabolic syndrome: A prospective cohort study of mediation by socioeconomic factors and physical activity

**DOI:** 10.1097/MD.0000000000046838

**Published:** 2026-01-02

**Authors:** Xiaohong Lin, Yannv Qu

**Affiliations:** a Reproductive Health Care Department, Shenzhen Longhua Maternity and Child Healthcare Hospital, Shenzhen, China; b Geriatrics Department, Peking University Shenzhen Hospital, Shenzhen Peking University-The Hong Kong University of Science and Technology Medical Center, Shenzhen, China; c Medical School Department, Shenzhen University, Shenzhen, Guangdong, China.

**Keywords:** all-cause mortality, cardiovascular mortality, cardiovascular-kidney-metabolic syndrome, National Health and Nutrition Examination Survey, PHQ-9, physical activity, poverty income ratio, social determinants of health

## Abstract

Depression is prevalent in cardiovascular-kidney-metabolic (CKM) syndrome, but its complex association with mortality remains incompletely characterized. This prospective cohort study utilized National Health and Nutrition Examination Survey data (2007–2016), including 27,673 adults with CKM syndrome (median follow-up 93.5 months). Associations between patient health questionnaire-9 (PHQ-9) scores and all-cause/cardiovascular mortality were analyzed using multivariable Cox regression, threshold models, subgroup, and mediation analyses. Among 2468 all-cause and 745 cardiovascular deaths, deceased individuals were significantly older, more frequently male, and more often non-Hispanic White. Higher CKM stage (stages 3–4: 63.9% of deaths vs 7.8% survivors), clinical parameters, and lower socioeconomic status predicted mortality (all *P* < .001). PHQ-9 demonstrated a non-linear, J-shaped association with mortality. Per 1-point PHQ-9 increase, fully adjusted all-cause mortality risk rose 1% (hazard ratio (HR) = 1.01, 95% confidence interval (CI): 1.00–1.02, *P* = .030). Cardiovascular mortality association was non-significant after full adjustment (HR = 1.02, 95% CI: 1.00–1.04, *P* = .076). A significant inflection point occurred at PHQ-9 = 11. Below 11, each point increase significantly elevated all-cause (HR = 1.03, 95% CI: 1.01–1.04, *P* = .0001) and cardiovascular mortality risk (HR = 1.05, 95% CI: 1.02–1.08, *P* = .0004). Above 11, associations were non-significant. The PHQ-9-mortality association was significantly stronger in participants ≤ 60 years (*P*-interaction = .001), with moderate/high chronic kidney disease risk (*P*-interaction = .029), and with metabolic syndrome (*P*-interaction = .024). Physical activity, poverty income ratio, and marital status were found to significantly account for a portion (12.76–14.80%) of the association between PHQ-9 scores and all-cause mortality (all *P* < .0001). Depressive symptoms demonstrate threshold-specific mortality risks in CKM syndrome. Socioeconomic factors (income, marital status) and physical activity significantly attenuate depression-associated mortality risk, partly mediating the association.

**Key Points**

1. Non-linear mortality link: PHQ-9 depression severity exhibits a J-shaped association with mortality in CKM syndrome, with significant risk escalation below a threshold of PHQ-9 = 11 (all-cause: HR = 1.03 per point, *P* < .001; CVD: HR = 1.05, *P* < .001).
2. Critical Effect Modifiers: The PHQ-9–mortality association is significantly stronger in younger patients (≤60 years), those with moderate/high CKD risk, or MetS (all *P*-interaction < .05).
3. Socioeconomic-physical mediation: Physical activity, higher income, and marriage explained 12.76% to 14.80% of the association between depressive symptoms and mortality risk.
4. Stage-driven outcomes: Advanced CKM stages (3–4) account for 63.9% of deaths versus 7.8% in survivors.

## 1. Introduction

Cardiovascular-kidney-metabolic (CKM) syndrome, defined by the American Heart Association as the interplay of cardiovascular disease (CVD), chronic kidney disease (CKD), and metabolic disorders (e.g., obesity and diabetes), represents a complex public health challenge requiring urgent attention.^[1]^ Epidemiological studies reveal that nearly 90% of US adults meet criteria for CKM stages 1 to 3, with 9.2% in high-risk stage 4,^[2]^ underscoring its high prevalence and the critical need for preventive interventions given the elevated morbidity and mortality associated with advanced disease. While established biological mechanisms such as chronic inflammation, oxidative stress, and metabolic dysregulation contribute to CKM progression,^[1,3]^ the role of social determinants of health remains underexplored.

Concurrently, depression – a highly prevalent mental illness affecting approximately 280 million people globally – constitutes the leading cause of mental health-related disability, accounting for 37.3% of all mental disorder disability-adjusted life years with rising prevalence trends.^[4,5]^ Beyond its disability burden, depression independently elevates mortality risk in the general population. Prospective cohort studies demonstrate a dose-response relationship, where mild depressive symptoms increase all-cause mortality by 35% and moderate-to-severe symptoms by 62% (National Health and Nutrition Examination Survey [NHANES] 2005–2018, n = 23,694).^[6]^ This association extends to diverse populations, both the China Kadoorie Biobank (n = 512,712) and Dongfeng-Tongji cohort (n = 26,298) reported 17 to 32% higher all-cause and cardiovascular mortality risks among depressed individuals.^[7]^

Critically, depression amplifies mortality risk when comorbid with chronic diseases. In type 2 diabetes (T2DM), depression confers a 36% increase in all-cause mortality (hazard ratio (HR) = 1.36).^[8,9]^ Similarly, major depression significantly exacerbates cardiovascular prognosis.^[10,11]^ Meta-analysis of 1.96 million subjects confirms depression as an independent predictor of adverse cardiovascular outcomes. Beyond “meta-analysis of 1.96 million subjects confirms depression as an independent predictor of adverse cardiovascular outcomes. Beyond elevating incident MI risk by 28% (HR = 1.28) and stroke risk by 13% (HR = 1.13), it disproportionately increases mortality – particularly CHF-related deaths (HR = 3.20) – underscoring its role in accelerating end-stage disease progression.^[10]^

Despite abundant evidence demonstrating a link between depression and mortality, a detailed and comprehensive analysis considering multiple mediators and confounding factors for this association is lacking. Current evidence inadequately characterizes the relationship between CKM health and depression, particularly regarding depression’s role as comorbidity in chronic disease progression. Critical gaps persist concerning the association of depression with all-cause and cardiovascular mortality in CKM patients, including potential score-response relationships and clinical thresholds. This study therefore investigates depression’s association with both all-cause and cardiovascular mortality in CKM syndrome while elucidating underlying pathophysiological mechanisms.

## 2. Methods

### 2.1. Data sources and study population

This study utilized data from the NHANES (2007–2016), a nationally representative cross-sectional survey designed to assess health and nutritional status in the United States. NHANES employs a stratified multistage probability sampling design to ensure population representativeness. Detailed methodology and datasets are publicly accessible via the centers for disease control and prevention website (https://www.cdc.gov/nchs/nhanes/).

**Figure 1.**
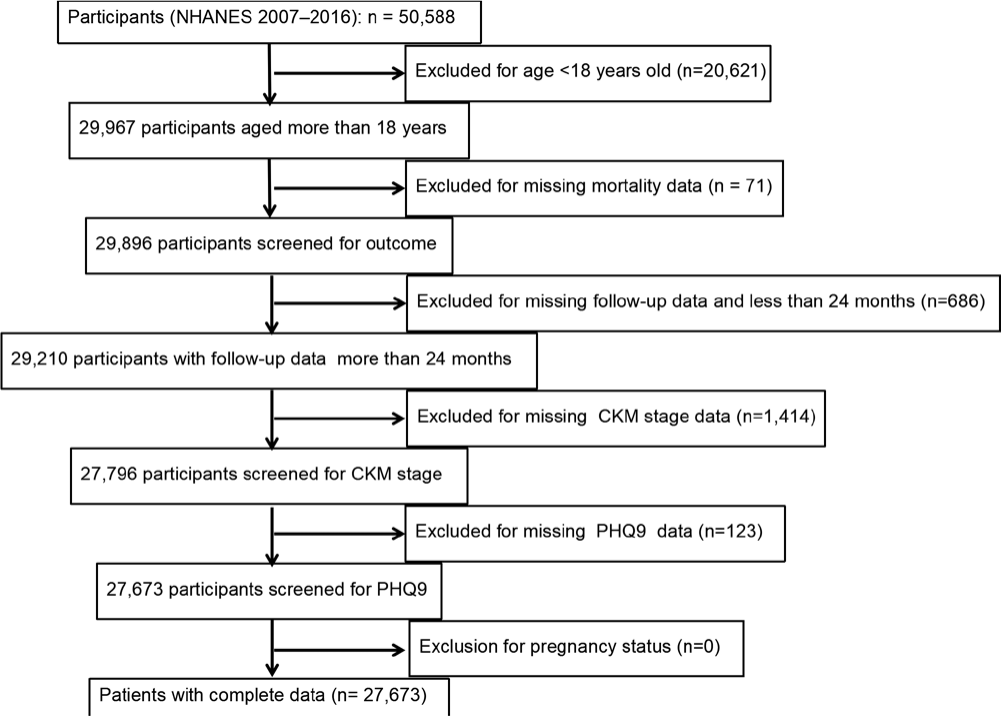
Flowchart of study population. CKM = cardiovascular-kidney-metabolic, NHANES = National Health and Nutrition Examination Survey, PHQ = patient health questionnaire.

In this prospective cohort study, NHANES data from 2007 to 2016 were analyzed, covering 5 two-year cycles. Exclusion criteria were applied to ensure the robustness of the results. From the initial dataset of 50,588 participants, 20,621 were excluded due to being aged 18 and under, leaving 29,967 participants. Then, 71 participants with missing mortality data were excluded, resulting in 29,896 participants. Subsequently, 686 participants with incomplete follow-up data or a follow-up duration of <24 months were excluded, leaving 29,210 participants. Then, 1414 participants with missing CKM stage data were excluded, leaving 27,796 participants. Finally, 123 participants with missing depression (PHQ9 – total) data were excluded, resulting in a final study population of 27,673 participants. None were excluded for pregnancy status. Throughout the screening process, each step was strictly carried out according to the set criteria to ensure the quality of the study sample and the reliability of the results (Fig. 1).

### 2.2. CKM syndrome stages 0 to 4

CKM syndrome was classified into stages 0 to 4 to capture the spectrum of cardiovascular, renal, and metabolic dysfunction: Stage 0 (no CKM risk factors); Stage 1 (excess adiposity, body mass index (BMI) ≥ 25 kg/m^2^); Stage 2 (metabolic syndrome (MeTS) [≥2 of hypertension, dyslipidemia, hyperglycemia] or moderate-to-high CKD risk [kidney disease improving global outcomes (KDIGO) criteria: estimated glomerular filtration rate (eGFR) = 30–59 mL/min/1.73 m^2^ or urinary albumin/creatinine ratio (UACR) ≥ 30 mg/g]); Stage 3 (subclinical CVD [10-year CVD risk ≥ 20% via predicting risk of CVD events equation^[12,13]^ or very high-risk CKD (eGFR < 30 mL/min/1.73 m^2^ or UACR ≥ 300 mg/g)]), and Stage 4 (clinically evident CVD, e.g., heart failure, stroke). CKD staging followed KDIGO guidelines,^[14]^ with eGFR calculated using the 2021 race-agnostic CKD-EPI equation^[15]^ (full criteria in Tables S1 and S2, Supplemental Digital Content, https://links.lww.com/MD/R42).

### 2.3. Mortality

The primary outcome, all-cause mortality (ICD-10: A00–Z99), and secondary outcome, cardiovascular mortality (heart disease: I00–I09, I11, I13, I20–I51; cerebrovascular disease: I60–I69), were ascertained through linkage to the National Death Index (NDI, updated December 31, 2019) via probabilistic matching (https://www.cdc.gov/nchs/data-linkage/mortality-public.htm).

### 2.4. Depression

Depression severity was assessed using the 9-item patient health questionnaire (PHQ-9), a validated instrument for screening clinically significant depression.^[16]^ Each item is scored 0 (“not at all”) to 3 (“nearly every day”), yielding a total score of 0 to 27. A cutoff score of ≥10, demonstrating 88% sensitivity and specificity for major depression diagnosis,^[16]^ was used to define cases.

### 2.5. Covariate

Demographic, clinical, and laboratory data were collected, including age, sex, race/ethnicity, education, marital status, poverty-income ratio, anthropometrics (BMI, waist circumference, height), and biochemical parameters (eGFR [calculated via CKD-EPI 2021 equation], UACR, uric acid, fasting glucose, hemoglobin A1c (HbA1c), triglycerides (TGs), total cholesterol, high-density lipoprotein cholesterol [HDL-C], low-density lipoprotein cholesterol). Lifestyle factors (smoking, alcohol use, physical activity [≥150 minutes moderate or 75 minutes vigorous/week]) and medication use (antihypertensives, glucose-lowering agents, statins) were documented. Hypertension was defined as diagnosed disease or current antihypertensive treatment. Metabolic syndrome required ≥ 3 criteria: waist circumference ≥ 102 cm (men)/≥88 cm (women), HDL-C < 40 mg/dL (men)/<50 mg/dL (women), TGs ≥ 150 mg/dL, or fasting glucose ≥ 100 mg/dL. Diabetes was confirmed by fasting glucose > 126 mg/dL, HbA1c ≥ 6.5%, clinical diagnosis, or glucose-lowering therapy. CKD risk stratification followed KDIGO guidelines (eGFR and UACR-based low/moderate/high risk).^[14]^ Standardized protocols for all measurements are described at Table S3 (Supplemental Digital Content, https://links.lww.com/MD/R42).

### 2.6. Statistical analyzes

Continuous variables are summarized as mean ± standard deviation, and categorical variables as frequencies (percentages). Participants were stratified by survival status (all-cause and cardiovascular mortality) for baseline comparisons, with group differences assessed via one-way ANOVA (continuous variables) or χ^2^ tests (categorical variables).

To evaluate non-linear associations between PHQ-9 score and mortality, generalized additive models were applied, incorporating smoothing splines to identify potential threshold effects. Cox proportional hazards regression models generated HRs with 95% confidence intervals (CIs), adjusted for covariates selected via clinical relevance and >10% effect size changes: age; gender; race; education level; marital status; poverty income ratio (PIR); smoking; drinking; physical activity; BMI; blood urea nitrogen; HBA1c; FBS; eGFR; hypertension; CVD; antihypertensive agents; antihyperglycemic agents; CKD Risk; MeTS; CKM syndrome (Fig. S4, Supplemental Digital Content, https://links.lww.com/MD/R42). Missing data were addressed using dummy variables^[17]^ (Table S15, Supplemental Digital Content, https://links.lww.com/MD/R42). Threshold effects were examined via two-piecewise linear regression, iteratively testing knots across the PHQ-9 score range to identify the optimal inflection point (maximized likelihood) (Supplementary Appendix 2 R code for threshold, Supplemental Digital Content, https://links.lww.com/MD/R42). To compare the linear and non-linear (threshold) models, we employed the LRT, which is appropriate for comparing nested models in the context of association analysis.

To assess the robustness of our primary findings, we conducted a series of comprehensive sensitivity analyses. First, to address potential reverse causation, we repeated the main analyses after sequentially excluding all mortality cases that occurred within the first 3 and 5 years of follow-up, respectively. Second, we employed different strategies to handle missing data in the covariates. In addition to creating a dummy variable category for responses such as “do not know,” “prefer not to answer,” or missing, we also performed a multiple imputation procedure to generate complete datasets.^[18]^ The results from models using the dummy variable approach and the multiple imputation approach were compared with the primary analysis.

Third, we calculated *E*-values to quantify the potential impact of unmeasured confounding on the observed association between PHQ-9 scores and mortality.^[19]^ The *E*-value represents the minimum strength of association that an unmeasured confounder would need to have with both the exposure and the outcome, beyond the measured covariates, to fully explain the observed HR. Finally, we examined possible effect modification by conducting stratified Cox regression analyses across key subgroups, including gender, age groups, and history of specific diseases and medications. The presence of statistical interaction was formally tested using LRTs.

Exploratory analysis of potential pathway variables in the PHQ-9 and mortality association were tested using the R “mediation” package (v4.4.3), adjusting for the aforementioned covariates. Statistical significance was defined as two-sided *P* < .05. Analyses were performed using EmpowerStats (http://www.empowerstats.com) and R (v4.4.3; http://www.r-project.org).

## 3. Results

Over a median follow-up of 93.5 months, 2468 all-cause and 745cardiovascular deaths occurred among 27,673 participants with CKM syndrome in NHANES (2007–2016). Compared to survivors, those who died from all causes (n = 2468) and cardiovascular causes (n = 745) were significantly older (69.5 ± 12.3 vs 46.4 ± 16.9 years and 71.3 ± 11.5 vs 47.7 ± 17.5 years, respectively) and more frequently male (57.4% vs 48.5%; 57.8% vs 49.0%). Racial disparities emerged, with non-Hispanic White individuals disproportionately represented among fatalities (61.6% vs 40.2% in all-cause deaths). While clinical parameters (glycemic control, renal function) and comorbidities (diabetes, CVD) showed significant mortality-associated gradients, socioeconomic factors including education and income levels also demonstrated mortality-linked stratification (all *P* < .001). Advanced CKM stages (3–4) accounted for 63.9% of deaths versus 7.8% in survivors (Table 1, Tables S4 and S5, Supplemental Digital Content, https://links.lww.com/MD/R42). Variables with missing data exceeding 5% were systematically addressed using dummy variables to account for missingness in statistical analyses. Table S6 (Supplemental Digital Content, https://links.lww.com/MD/R42) demonstrates that participants with depression exhibited significantly higher BMI, TGs, waist circumference, prevalence of metabolic syndrome (MetS), CVD, stroke, low education levels, and all-cause mortality compared to non-depressed individuals. Conversely, serum vitamin D levels, physical activity, and antihypertensive/lipid-lowering medication use were notably lower. No significant differences were observed in age, eGFR, or UACR between groups. Table S7 (Supplemental Digital Content, https://links.lww.com/MD/R42) revealed significant stage-dependent disparities across CKM stages (0–4) in metabolic parameters (BMI, eGFR, UACR), cardiometabolic comorbidities (hypertension, MetS), medication utilization, and mortality outcomes, with all critical biomarkers and clinical indicators showing statistically significant differences (*P* < .001).

**Table 1:** Baseline characteristics of patients with CKM syndrome stages 0–4 concerning all-cause mortality and cardiovascular mortality (N = 27,673).

		All-cause mortality			Cardiovascular mortality		
		Survivors	Deceased	*P*-value	Survivors	Deceased	*P*-value
Age, yr	48.3 ± 17.7	46.4 ± 16.9	69.5 ± 12.3	<.001	47.7 ± 17.5	71.3 ± 11.5	<.001
Gender, n (%)							
Male	13,430 (48.5%)	12,057 (47.8%)	1373 (55.6%)	<.001	13,007 (48.3%)	423 (56.8%)	<.001
Female	14,243 (51.5%)	13,148 (52.2%)	1095 (44.4%)		13,921 (51.7%)	322 (43.2%)	
Race, n (%)							
Mexican American	4359 (15.8%)	4157 (16.5%)	202 (8.2%)	<.001	4310 (16.0%)	49 (6.6%)	<.001
Other Hispanic	3018 (10.9%)	2847 (11.3%)	171 (6.9%)		2963 (11.0%)	55 (7.4%)	
Non-Hispanic White	11,326 (40.9%)	9858 (39.1%)	1468 (59.5%)		10,872 (40.4%)	454 (60.9%)	
Non-Hispanic Black	5915 (21.4%)	5408 (21.5%)	507 (20.5%)		5757 (21.4%)	158 (21.2%)	
Other Race - Including Multi-Racial	1077 (3.9%)	1002 (4.0%)	75 (3.0%)		1057 (3.9%)	20 (2.7%)	
Non-Hispanic Asian	1978 (7.1%)	1933 (7.7%)	45 (1.8%)		1969 (7.3%)	9 (1.2%)	
Education level, n (%)							
<High School grade	6909 (25.0%)	6017 (23.9%)	892 (36.1%)	<.001	6630 (24.6%)	279 (37.4%)	<.001
High School grade/GED or Equivalent	6105 (22.1%)	5479 (21.7%)	626 (25.4%)		5913 (22.0%)	192 (25.8%)	
Some College or above	13,959 (50.4%)	13,018 (51.6%)	941 (38.1%)		13,687 (50.8%)	272 (36.5%)	
Marital status, n (%)							
Married or Living with partner	16,069 (58.1%)	14,846 (58.9%)	1223 (49.6%)	<.001	15,716 (58.4%)	353 (47.4%)	<.001
Separated or Never married	10,918 (39.5%)	9677 (38.4%)	1241 (50.3%)		10,526 (39.1%)	392 (52.6%)	
Poverty income ratio, n (%)							
≤1.30	9546 (34.5%)	8631 (34.2%)	915 (37.1%)	<.001	9291 (34.5%)	255 (34.2%)	<.001
1.3~1.85	3774 (13.6%)	3351 (13.3%)	423 (17.1%)		3629 (13.5%)	145 (19.5%)	
> 1.85	12,579 (45.5%)	11,608 (46.1%)	971 (39.3%)		12,277 (45.6%)	302 (40.5%)	
Smoking, n (%)							
Never	15,287 (55.2%)	14,301 (56.7%)	986 (40.0%)	<.001	14,940 (55.5%)	347 (46.6%)	<.001
Current smoker	5581 (20.2%)	5089 (20.2%)	492 (19.9%)		5463 (20.3%)	118 (15.8%)	
Ever smoker	6357 (23.0%)	5372 (21.3%)	985 (39.9%)		6077 (22.6%)	280 (37.6%)	
Drinking, g/yr	40.2 ± 392.8	36.6 ± 254.1	95.0 ± 1231.9	.581	39.7 ± 384.8	68.1 ± 688.8	<.001
FBS, mg/dL	109.3 ± 36.7	107.9 ± 34.7	123.7 ± 50.9	<.001	108.9 ± 36.3	123.4 ± 45.9	<.001
HBA1c, %	5.8 ± 1.1	5.7 ± 1.0	6.2 ± 1.3	<.001	5.8 ± 1.1	6.2 ± 1.4	<.001
TC, mg/dL	192.7 ± 41.8	193.1 ± 41.1	188.2 ± 44.6	<.001	192.9 ± 41.6	186.8 ± 45.3	.004
LDL-C, mg/dL	113.6 ± 35.4	114.3 ± 35.1	106.3 ± 37.7	<.001	113.8 ± 35.3	105.8 ± 39.0	<.001
HDL-C, mg/dL	52.5 ± 15.9	52.6 ± 15.9	52.7 ± 17.7	.919	52.7 ± 16.1	51.9 ± 16.4	.244
TG, mg/dL	155.5 ± 134.4	155.3 ± 136.5	158.0 ± 109.6	.358	155.6 ± 135.2	155.1 ± 96.7	.922
eGFR, mL/min/1.73 m^2^	95.6 ± 22.7	97.8 ± 21.4	73.1 ± 23.7	<.001	96.4 ± 22.2	69.2 ± 22.8	<.001
Standing height, cm	167.0 ± 10.2	167.2 ± 10.2	165.8 ± 10.4	<.001	167.1 ± 10.2	165.2 ± 10.2	<.001
Waist circumference, cm	99.2 ± 16.2	98.9 ± 16.1	103.0 ± 16.0	<.001	99.1 ± 16.2	103.9 ± 15.8	<.001
BMI, kg/m^2^	29.2 ± 6.8	29.2 ± 6.8	29.1 ± 6.8	.334	29.2 ± 6.8	29.4 ± 7.0	.349
PHQ9 score	2.9 ± 4.3	2.9 ± 4.2	3.2 ± 4.4	.003	2.9 ± 4.3	3.2 ± 4.3	.040
Physical activity, n (%)	14,489 (52.4%)	13,742 (54.5%)	747 (30.3%)	<.001	14,268 (53.0%)	221 (29.7%)	<.001
Diabetes, n (%)	3442 (12.4%)	2744 (10.9%)	698 (28.3%)	<.001	3207 (11.9%)	235 (31.5%)	<.001
Hypertension, n (%)	9703 (35.1%)	8094 (32.1%)	1609 (65.2%)	<.001	9186 (34.1%)	517 (69.4%)	<.001
Cardiovascular disease, n (%)	2166 (7.8%)	1498 (5.9%)	668 (27.1%)	<.001	1901 (7.1%)	265 (35.6%)	<.001
Hyperlipidemia, n (%)	8914 (32.2%)	7743 (30.7%)	1171 (47.4%)	<.001	8528 (31.7%)	386 (51.8%)	<.001
Antihypertensive agents, n (%)	8427 (30.5%)	6896 (27.4%)	1531 (62.0%)	<.001	7927 (29.4%)	500 (67.1%)	<.001
Antihyperlipidemic agents, n (%)	6445 (23.3%)	5413 (21.5%)	1032 (41.8%)	<.001	6105 (22.7%)	340 (45.6%)	<.001
Antihyperglycemic agents, n (%)	2500 (72.6%)	2022 (73.7%)	478 (68.5%)	.006	2334 (72.8%)	166 (70.6%)	.478
CKD risk, n (%)							
Low-risk	24,162 (87.3%)	22,529 (89.4%)	1633 (66.2%)	<.001	23,709 (88.0%)	453 (60.8%)	<.001
Moderate to high-risk	3118 (11.3%)	2446 (9.7%)	672 (27.2%)		2876 (10.7%)	242 (32.5%)	
Very high-risk	393 (1.4%)	230 (0.9%)	163 (6.6%)		343 (1.3%)	50 (6.7%)	
MetS, n (%)	10,219 (36.9%)	9021 (35.8%)	1198 (48.5%)		9828 (36.5%)	391 (52.5%)	<.001
CKM_Stage, n (%)							
Stage 0	5560 (20.1%)	5377 (21.3%)	183 (7.4%)	<.001	5534 (20.6%)	26 (3.5%)	<.001
Stage 1	9324 (33.7%)	8997 (35.7%)	327 (13.2%)		9243 (34.3%)	81 (10.9%)	
Stage 2	9299 (33.6%)	8836 (35.1%)	463 (18.8%)		9187 (34.1%)	112 (15.0%)	
Stage 3	1172 (4.2%)	57 (0.2%)	1115 (45.2%)		771 (2.9%)	401 (53.8%)	
Stage 4	2318 (8.4%)	1938 (7.7%)	380 (15.4%)		2193 (8.1%)	125 (16.8%)	

Frequencies are expressed as absolute numbers and percentages (%); values are means (standard deviation). Among the 23,635 patients, the amount of missing values for the covariates were 12,123 (51.3%) for education level, 9134 (38.6%) for marital status, 10,911 (46.2%) for poverty income ratio, 349 (<0.1%) for smoking, 8885 (37.1%) for drinking, 11,385 (48.2%) for FBS, 11,049 (46.7%) for LDL-C. Numbers not totaling 100% are due to missing data. Dummy variables were used to indicate missing covariate values.

BMI = body mass index, BUN = blood urea nitrogen, CI = confidence interval, CKD = chronic kidney disease, CKM = cardiovascular-kidney-metabolic, DBP = diastolic blood pressure, DM = diabetes mellitus, eGFR = estimated glomerular filtration rate, FBG = fasting blood glucose, HbA1c = hemoglobin A1c, HDL-C = high-density lipoprotein cholesterol, HR = hazard ratio, LDL-C = low-density lipoprotein cholesterol, MetS = metabolic syndrome, TC = total cholesterol, TG = triglyceride, UA = uric acid, UACR = urinary albumin/creatinine ratio.

Among 27,673 participants, substantial missingness (>5%) was observed for fasting blood glucose (63.3%), low-density lipoprotein cholesterol (65.4%), PIR (7.5%), and key metabolic/renal markers: eGFR (7.1%), TGs (7.2%), creatinine (7.1%), blood urea nitrogen (7.1%). Moderate missingness affected waist circumference (5.9%), HbA1c (5.7%), HDL-C (6.8%), and total cholesterol (6.8%). Lower missing rates (<5%) occurred in education (3.0%), marital status (2.9%), UACR (2.3%), smoking (1.9%), BMI (0.7%), and standing height (0.6%). Numbers not total 100% are due to missing data. Variables with missing data exceeding 5% were systematically addressed using dummy variables and multiple imputation to account for missingness in statistical analyses.

### 3.1. Association between PHQ-9 score and mortality

**Figure 2.**
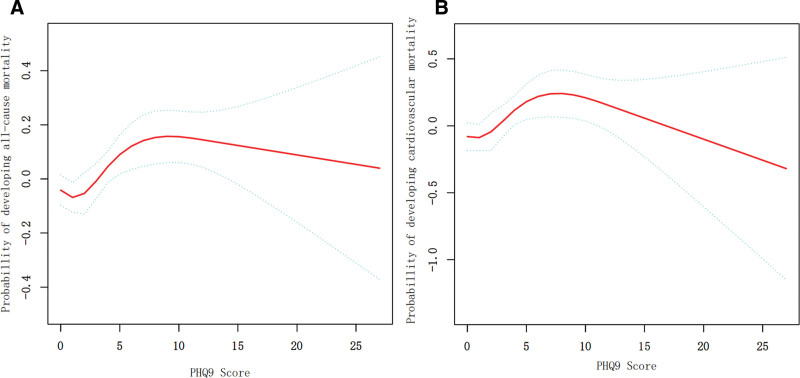
Association between PHQ9 score and all-cause (A) and cardiovascular mortality (B) in a group with CKM syndrome stage 0–4. CKM = cardiovascular-kidney-metabolic, PHQ = patient health questionnaire.

In the Cox model analysis, PHQ-9 score exhibited a non-linear relationship with mortality risk, where all-cause mortality risk initially rose with increasing scores, peaked around PHQ-9 = 10, and then declined slightly at higher values (Fig. 2). For all-cause mortality, the fully adjusted model (Adjust II) showed a significant 1% increase in risk per 1-point PHQ-9 increment (HR = 1.01, 95% CI: 1.00–1.02, *P* = .0302), while categorical analysis revealed non-significant trends: the 10 to 14 group had HR = 1.15 (95% CI: 0.95–1.39, *P* = .1544) and the ≥15 group had HR = 1.02 (95% CI: 0.80–1.31, *P* = .8629). For cardiovascular mortality, the continuous PHQ-9 association became non-significant in Adjust II (HR = 1.02, 95% CI: 1.00–1.04, *P* = .0760), with categorical groups similarly losing significance (10–14: HR = 1.37, 95% CI: 0.99–1.91, *P* = .0611; ≥15: HR = 0.89, 95% CI: 0.54–1.47, *P* = .6491) (Table 2).

**Table 2:** Cox proportional hazards regression analysis of PHQ9 score indices concerning all-cause and cardiovascular mortality in a CKM syndrome stage 0–4 population.

Exposure	Non-adjusted	Adjust I	Adjust II
All-cause mortality			
PHQ-9 score	1.01 (1.00, 1.02) 0.0070	1.05 (1.04, 1.05) <0.0001	1.01 (1.00, 1.02) 0.0302
PHQ-9 score categorical			
<10	Ref.	Ref.	Ref.
≥10, <15	1.11 (0.94, 1.32) 0.2288	1.69 (1.42, 2.01) <0.0001	1.15 (0.95, 1.39) 0.1544
≥15	1.22 (1.00, 1.50) 0.0525	1.87 (1.52, 2.29) <0.0001	1.02 (0.80, 1.31) 0.8629
Exposure	Non-adjusted	Adjust I	Adjust II
Cardiovascular mortality			
PHQ-9 score	1.02 (1.00, 1.03) 0.0499	1.06 (1.04, 1.07) <0.0001	1.02 (1.00, 1.04) 0.0760
PHQ-9 score categorical			
<10	Ref.	Ref.	Ref.
≥10, <15	1.26 (0.94, 1.69) 0.1179	2.08 (1.55, 2.80) <0.0001	1.37 (0.99, 1.91) 0.0611
≥15	1.00 (0.67, 1.51) 0.9839	1.68 (1.11, 2.54) 0.0132	0.89 (0.54, 1.47) 0.6491

Data were presented as HR (95% CI), *P* value; non-adjusted model adjust for: none.

Adjust I model adjust for: age; gender; race; Adjust II model adjust for: age; gender; race; education level; marital status; poverty income ratio; smoking; drinking; physical activity; BMI; BUN; HBA1c; FBS; eGFR; hypertension; cardiovascular disease; antihypertensive agents; antihyperglycemic agents; CKD Risk; MeTS; CKM syndrome.

BMI = body mass index, BUN = blood urea nitrogen, CI = confidence interval, CKD = chronic kidney disease, CKM = cardiovascular-kidney-metabolic, DBP = diastolic blood pressure, DM = diabetes mellitus, eGFR = estimated glomerular filtration rate, FBG = fasting blood glucose, HbA1c = hemoglobin A1c, HR = hazard ratio, MeTS = metabolic syndrome, Ref = reference, TG = triglyceride, UA = uric acid, UACR = urinary albumin/creatinine ratio.

### 3.2. Threshold effect analysis

In patients with CKM syndrome, PHQ-9 scores showed a non-linear relationship with mortality outcomes (Table 3). For all-cause mortality, Model I indicated a significant 2% risk increase per 1-point PHQ-9 rise (HR = 1.02, 95% CI: 1.01–1.03, *P* = .0005). Model II identified a turning point at PHQ-9 = 11 (95% CI: 7–12), with a significant 3% risk increase per point below this threshold (HR = 1.03, 95% CI: 1.01–1.04, *P* = .0001) and non-significant results above it (HR = 0.99, 95% CI: 0.95–1.02, *P* = .4376). The likelihood ratio test (LRT) *P* value was .054.

**Table 3:** Analysis of the threshold effect of PHQ9 score on all-cause and cardiovascular mortality in patients with CKM syndrome stage 0–4.

PHQ9 score Outcome:	All-cause mortality	Cardiovascular mortality
Model I		
One line effect	1.02 (1.01, 1.03) 0.0005	1.02 (1.00, 1.04) 0.0198
Model II		
Turning point (K)	11	11
<K	1.03 (1.01, 1.04) 0.0001	1.05 (1.02, 1.08) 0.0004
≥K	0.99 (0.95, 1.02) 0.4376	0.93 (0.86, 1.01) 0.0768
*P* value for LRT test	.054	.008
95% CI for turning point	7, 12	7, 12

Data were presented as HR (95% CI), *P* value; Model I, linear analysis; Model ll, non-linear analysis. Adjust for: age; gender; race; education level; marital status; poverty income ratio; smoking; drinking; physical activity; BMI; BUN; HBA1c; FBS; eGFR; hypertension; cardiovascular disease; antihypertensive agents; antihyperglycemic agents; CKD Risk; MeTS; CKM syndrome.

BMI = body mass index, BUN = blood urea nitrogen, CI = confidence interval, CKD = chronic kidney disease, CKM = cardiovascular-kidney-metabolic, DBP = diastolic blood pressure, DM = diabetes mellitus, eGFR = estimated glomerular filtration rate, FBG = fasting blood glucose, HbA1c = hemoglobin A1c, HR = hazard ratio, LRT = logarithm likelihood ratio test, MeTS = metabolic syndrome, Ref = reference, TG = triglyceride.

For cardiovascular mortality, Model I showed a non-significant 2% risk increase (HR = 1.02, 95% CI: 1.00–1.04, *P* = .0198). Model II also found a turning point at PHQ-9 = 11 (95% CI: 7–12), with scores below linked to a significant 5% risk increase (HR = 1.05, 95% CI: 1.02–1.08, *P* = .0004) and those above associated with a non-significant 7% risk reduction (HR = 0.93, 95% CI: 0.86–1.01, *P* = .0768). The LRT *P* value here was significant at .008.

### 3.3. Subgroup analysis

**Figure 3.**
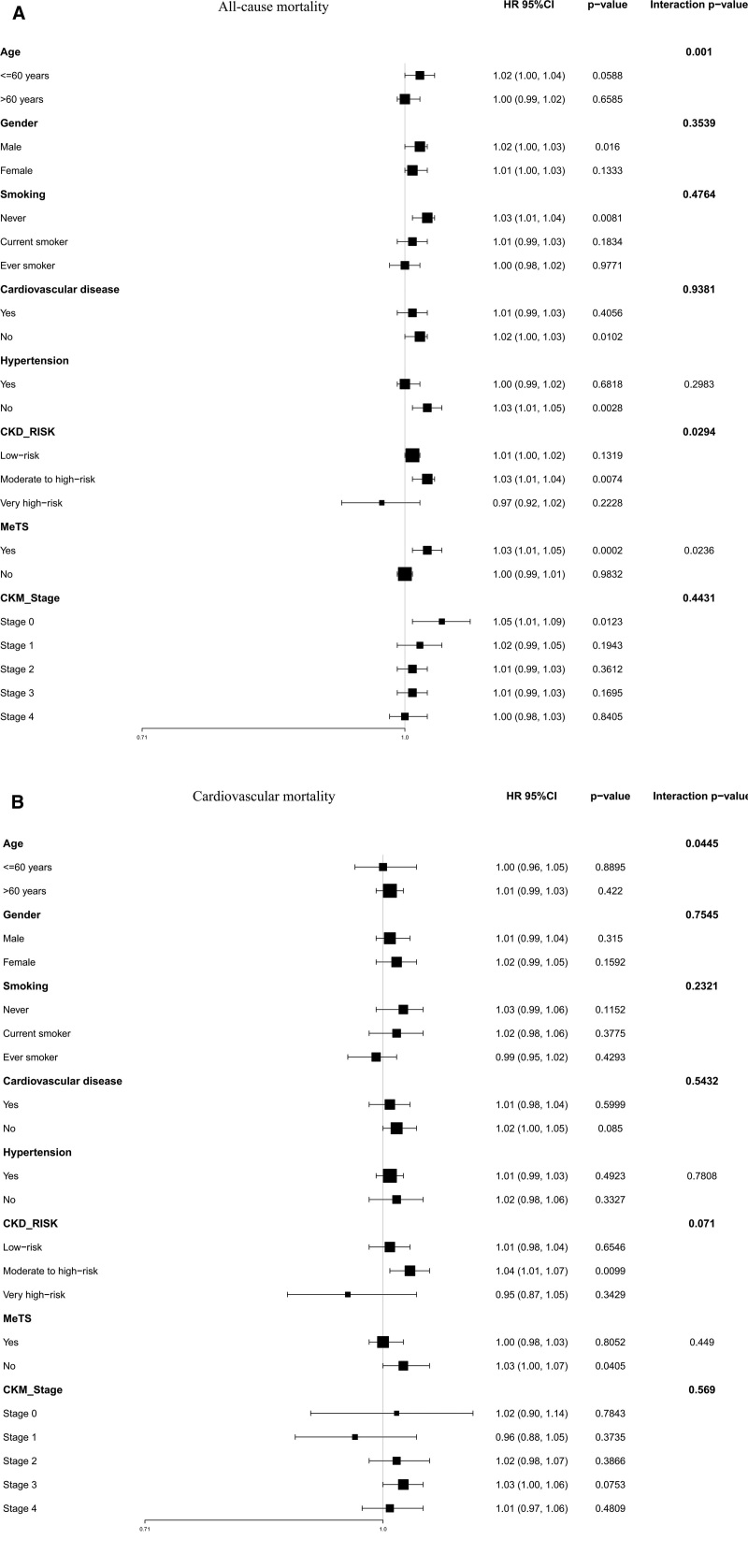
Subgroup analysis of PHQ9 score on all-cause (A) and cardiovascular mortality (B) in a group with CKM syndrome stage 0–4. CKM = cardiovascular-kidney-metabolic, PHQ = patient health questionnaire.

In subgroup analyses for all-cause mortality, age exhibited a significant interaction (*P*-interaction = .001), with elevated risk in participants ≤60 years (HR = 1.02, 95% CI: 1.00–1.04, *P* = .059) but no association in those >60 years (HR = 1.00, 95% CI: 0.99–1.02, *P* = .659). Significant effect modifications were also observed for CKD risk (moderate/high vs low: HR = 1.03, 95% CI: 1.01–1.04, *P* = .007; *P*-interaction = .029) and MeTS (yes vs no: HR = 1.03, 95% CI: 1.01–1.05, *P* < .001; *P*-interaction = .024). For cardiovascular mortality, age showed borderline interaction (*P*-interaction = .044), with non-significant trends across age strata (≤60: HR = 1.00, 95% CI: 0.96–1.05; >60: HR = 1.01, 95% CI: 0.99–1.03). CKD risk approached significance (moderate/high vs low: HR = 1.04, 95% CI: 1.01–1.07, *P* = .010; *P*-interaction = .071), while MeTS absence was unexpectedly associated with increased risk (HR = 1.03, 95% CI: 1.00–1.07, *P* = .041) despite non-significant interaction (*P*-interaction = .449). No interactions were detected for gender, smoking, hypertension, or CKM stage in either outcome (all *P*-interaction > .05) (Fig. 3).

### 3.4. Sensitivity analyses

#### 3.4.1. Unmeasured-confounding robustness

An *E*-value of 1.11 for the point estimate (HR = 1.01; 95% CI: 1.00–1.02) indicates that an unmeasured confounder would need to confer ≥11% excess risk for both higher PHQ-9 and all-cause mortality, conditional on all covariates already entered, to nullify the observed association; the lower-limit *E*-value is 1.00.

We also performed sensitivity analyses restricting the cohort to participants with follow-up durations >36 months and >60 months to assess the robustness of the primary findings. Cohorts with >36 months (n = 27,297; depressed 8.2%) and >60 months (n = 21,462; depressed 8.4%) reproduced the baseline profile of the main cohort: depressed participants were more often female, non-Hispanic Black, unmarried, less educated, poorer, physically inactive, and burdened by obesity, MetS, CKD, CVD, stroke, and adverse laboratory markers (all *P* < .001) (Tables S7 and S8, Supplemental Digital Content, https://links.lww.com/MD/R42).

Mortality associations remained directionally identical across follow-up windows. In fully-adjusted Cox models, each 1-point PHQ-9 increase predicted a 2% rise in all-cause mortality (HR = 1.02; 95% CI: 1.00–1.03, *P* = .0062 for >36 months; HR = 1.02; 95% CI: 1.01–1.03, *P* = .0066 for >60 months). Categorical analyses showed non-significant increments for scores 10 to 14 and ≥15 (Tables S9 and S10, Supplemental Digital Content, https://links.lww.com/MD/R42). Cardiovascular mortality tracked positively with PHQ-9 but did not reach significance (HR = 1.02; 95% CI: 0.99–1.04, *P* = .1961 and HR = 1.02; 95% CI: 1.00–1.04, *P* = .0693, respectively). Restricted cubic splines replicated the main nonlinear shape (Figs. S1 and S2, Supplemental Digital Content, https://links.lww.com/MD/R42).

To address potential bias from missing data, we repeated our primary analyses using multiple imputation. The results from these analyses were consistent with the main findings, confirming the robustness of the observed associations.

The analyses confirmed a non-linear relationship between PHQ-9 scores and mortality risk, identifying a consistent inflection point at a score of 11. The overall trends for both all-cause and cardiovascular mortality remained unchanged, with detailed results provided in the supplementary materials (Tables S11 and S12, Fig. S3, Supplemental Digital Content, https://links.lww.com/MD/R42). Table S13 (Supplemental Digital Content, https://links.lww.com/MD/R42) results of the threshold effect analysis remained consistent when multiple imputation was applied, confirming the robustness of the identified non-linear associations between PHQ-9 scores and mortality risks.

### 3.5. Associations of physical activity, poverty income ratio and marital status with depressive symptoms and mortality

#### 3.5.1. Associations with all-cause and cardiovascular mortality

As summarized in Table 4, several baseline variables showed significant statistical associations with mortality outcomes. Physical activity exhibited a protective association with both all-cause and cardiovascular mortality. For all-cause mortality, the HR was 0.38 (95% CI: 0.35–0.42) in the unadjusted analysis and 0.75 (95% CI: 0.68–0.83) in the fully adjusted model. A similar pattern was observed for cardiovascular mortality, with a fully adjusted HR = 0.79 (95% CI: 0.66–0.95).

**Table 4:** Cox regression of physical activity, poverty income ratio and marital status with all-cause and cardiovascular mortality.

Exposure	Non-adjusted	Adjust I	Adjust II
All-cause mortality			
Physical activity			
No	Ref.	Ref.	Ref.
Yes	0.38 (0.35, 0.42) <0.0001	0.56 (0.52, 0.62) <0.0001	0.75 (0.68, 0.83) <0.0001
Poverty income ratio			
≤1.30	Ref.	Ref.	Ref.
1.3–1.85	1.17 (1.05, 1.32) 0.0061	0.82 (0.73, 0.92) 0.0009	0.94 (0.83, 1.07) 0.3658
>1.85	0.79 (0.72, 0.87) <0.0001	0.56 (0.51, 0.61) <0.0001	0.74 (0.67, 0.83) <0.0001
Marital status			
Married or living with partner	Ref.	Ref.	Ref.
Separated or never married	1.53 (1.41, 1.66) <0.0001	1.54 (1.41, 1.67) <0.0001	1.26 (1.14, 1.38) <0.0001
Cardiovascular mortality			
Physical activity			
No	Ref.	Ref.	Ref.
Yes	0.37 (0.32, 0.43) <0.0001	0.58 (0.49, 0.68) <0.0001	0.79 (0.66, 0.95) 0.0114
Poverty income ratio			
≤1.30	Ref.	Ref.	Ref.
1.3–1.85	1.45 (1.18, 1.77) 0.0004	0.97 (0.79, 1.19) 0.7396	1.15 (0.92, 1.45) 0.2178
>1.85	0.89 (0.75, 1.05) 0.1521	0.61 (0.52, 0.73) <0.0001	0.84 (0.69, 1.03) 0.0970
Marital status			
Married or living with partner	Ref.	Ref.	Ref.
Separated or never married	1.67 (1.45, 1.93) <0.0001	1.65 (1.41, 1.92) <0.0001	1.37 (1.15, 1.62) 0.0004

Data were presented as HR (95% CI), *P* value; Model I, linear analysis; Model II, non-linear analysis. Adjust for: age; gender; race; education level; marital status; poverty income ratio; smoking; drinking; physical activity; BMI; BUN; HBA1c; FBS; eGFR; hypertension; cardiovascular disease; antihypertensive agents; antihyperglycemic agents; CKD Risk; MeTS; CKM syndrome.

BMI = body mass index, BUN = blood urea nitrogen, CI = confidence interval, CKD = chronic kidney disease, CKM = cardiovascular-kidney-metabolic, DBP = diastolic blood pressure, DM = diabetes mellitus, eGFR = estimated glomerular filtration rate, FBG = fasting blood glucose, HbA1c = hemoglobin A1c, HR = hazard ratio, MeTS = metabolic syndrome, Ref = reference.

A higher PIR > 1.85 was associated with lower all-cause mortality risk (adjusted HR = 0.74, 95% CI: 0.67–0.83) compared to the lowest income group (PIR ≤ 1.30). For cardiovascular mortality, the association was in the same direction but did not reach statistical significance (adjusted HR = 0.84, 95% CI: 0.69–1.03). Individuals who were separated or never married showed higher mortality risks compared to those who were married or cohabiting, for both all-cause (adjusted HR = 1.26, 95% CI: 1.14–1.38) and cardiovascular mortality (adjusted HR = 1.37, 95% CI: 1.15–1.62).

#### 3.5.2. Associations with depressive symptoms

The relationships between these variables and PHQ-9 scores are presented in Table 5. Higher physical activity levels were associated with lower depression scores in both unadjusted (β = −0.55, 95% CI: −0.60 to −0.50) and fully adjusted analyses (β = −0.65, 95% CI: −0.73 to −0.59). Similarly, higher PIRs were associated with fewer depressive symptoms. Compared to the lowest income group, both the middle-income (PIR 1.3–1.85; adjusted β = −0.75, 95% CI: −0.86 to −0.65) and highest income groups (PIR > 1.85; adjusted β = −0.47, 95% CI: −0.53 to −0.42) showed significant negative associations. Additionally, being separated or never married was associated with higher depression scores (adjusted β = 1.57, 95% CI: 1.42–1.74) compared to being married or cohabiting.

**Table 5:** Linear regression of physical activity, poverty income ratio and marital status with depression.

Exposure	Non-adjusted	Adjust I	Adjust II
Physical activity			
No	Ref.	Ref.	Ref.
Yes	0.55 (0.50, 0.60) <0.0001	0.55 (0.50, 0.61) <0.0001	0.65 (0.59, 0.73) <0.0001
Poverty income ratio			
≤1.30	Ref.	Ref.	Ref.
1.3–1.85	0.62 (0.54, 0.71) <0.0001	0.64 (0.56, 0.73) <0.0001	0.75 (0.65, 0.86) <0.0001
>1.85	0.34 (0.31, 0.38) <0.0001	0.33 (0.30, 0.37) <0.0001	0.47 (0.42, 0.53) <0.0001
Marital status			
Married or living with partner	Ref.	Ref.	Ref.
Separated or never married	1.93 (1.77, 2.10) <0.0001	1.99 (1.82, 2.19) <0.0001	1.57 (1.42, 1.74) <0.0001

Data were presented as HR (95% CI), *P* value; Model I, linear analysis; Model II, non-linear analysis. Adjust for: age; gender; race; education level; marital status; poverty income ratio; smoking; drinking; physical activity; BMI; BUN; HBA1c; FBS; eGFR; hypertension; cardiovascular disease; antihypertensive agents; antihyperglycemic agents; CKD Risk; MeTS; CKM syndrome.

BMI = body mass index, BUN = blood urea nitrogen, CI = confidence interval, CKD = chronic kidney disease, CKM = cardiovascular-kidney-metabolic, eGFR = estimated glomerular filtration rate, HbA1c = hemoglobin A1c, HR = hazard ratio, MeTS = metabolic syndrome, Ref = reference.

#### 3.5.3. Exploration of pathway variables

**Figure 4.**
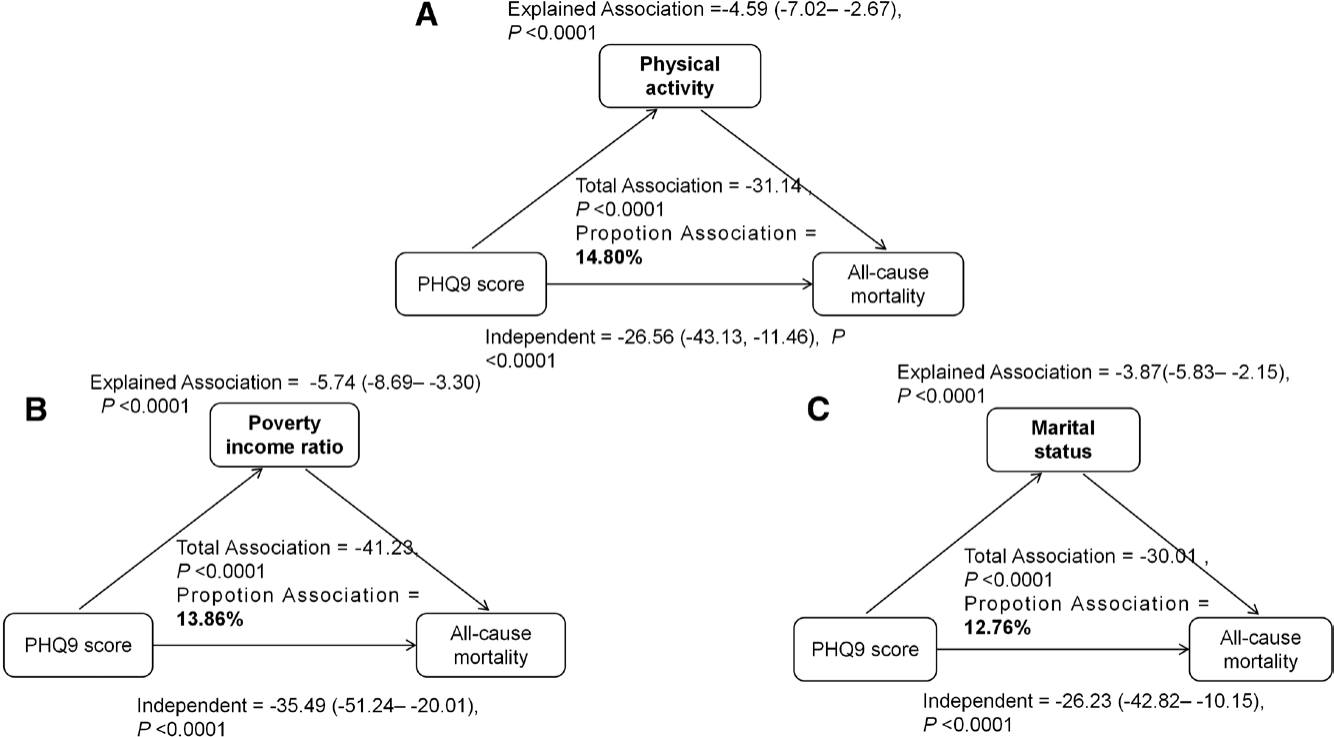
Exploratory analysis of physical activity (A) and poverty income ratio (B), marital status (C) in the PHQ-9 and mortality association. PHQ = patient health questionnaire.

We conducted an exploratory analysis to examine the extent to which the statistical association between PHQ-9 scores and all-cause mortality could be apportioned to several baseline pathway variables, including physical activity, PIR, and marital status. All 3 variables significantly explained a portion of the observed association (all *P* < .0001), with the proportions ranging from 12.76 to 14.80% (Fig. 4). Detailed results, including total, explained, and independent associations, are provided in Table S14 (Supplemental Digital Content, https://links.lww.com/MD/R42).

## 4. Discussion

In this national cohort of 27,673 patients with CKM syndrome, depression severity (PHQ-9) demonstrates a non-linear, threshold-dependent relationship with mortality, where risk significantly escalates below a score of 11. Compared with participants in stage 0, advanced CKM stages (3–4) drive >80% of deaths, underscoring disease severity as the primary mortality determinant. This association is potentiated in younger individuals (≤60 years), those with moderate/high CKD risk, or MetS. In an exploratory analysis, the factors of physical activity, higher income, and marital partnership were found to explain a significant proportion (12.76–14.80%) of the statistical association between depression and mortality.

Our findings align with previous literature linking depression to elevated medical comorbidities and mortality. Major depressive disorder is established as a risk factor for multiple chronic illnesses, including CVD, diabetes, stroke.^[7,8,11,20,21]^ In our study, we observed a non-linear pattern, where mortality risk increased with PHQ-9 scores up to a point and appeared to attenuate at the highest levels. In our study, we observed a non-linear pattern, where mortality risk increased with PHQ-9 scores up to a point. Although the point estimates suggested a potential plateauing of risk at the highest severity levels (PHQ-9 ≥ 15), this finding should be interpreted with caution due to the limited subgroup size and consequent wide CIs, which indicate insufficient statistical power to draw firm conclusions about the exact shape of the dose-response relationship at the extreme end. Emerging evidence from Chinese cohorts corroborates our findings. Gong et al (2022) demonstrated that individuals with ≥3 cardiometabolic comorbidities had over twice the odds of depression (OR 2.13),^[22]^ while longitudinal data from the China Health and Retirement Longitudinal Study indicate that cardiometabolic multimorbidity correlates with increasing risk of depressive symptoms.^[23]^

Our findings present an interesting contrast with the earlier work by Wang et al.^[24]^ Specifically, whereas their study reported a linear association between PHQ-9 scores and mortality risk in individuals with CKM stages 1 to 3 but no significant effect in stage 4. our analysis points to a nonlinear, J-shaped relationship. In our cohort, the mortality risk increased with rising PHQ-9 scores up to a threshold of 11, after which the risk appeared to attenuate. Another point of difference lies in the subgroup analyses. Unlike the previous report which identified effect modification by CKM stage, our results did not show a significant modifying effect of the CKM stage. The particularly strong association observed in younger adults underscores the potential clinical importance of depression screening in this subpopulation, even as we acknowledge that our analytical cohort may underrepresent healthier young adults, limiting the direct transportability of this finding to the general population. Consequently, the nature of this relationship – whether linear or nonlinear – and its modifiers should be a priority for investigation in future, more diverse cohorts.

### 4.1. Mechanistic considerations: multilevel pathways and a self-reinforcing cycle

The observed association between depression and higher mortality in cardiorenal‑metabolic (CKM) syndrome is consistent with contributions from biological, behavioral, and social factors that co‑occur and may interact. The following summarizes these multilevel pathways while avoiding causal claims.

Depression is associated with several biological alterations reported in the literature, including neurotransmitter dysregulation,^[25]^ neuroendocrine dysregulation (HPA axis hyperactivation) – leading to sustained hypercortisolemia,^[13]^ systemic inflammation (elevated proinflammatory cytokines),^[26]^ metabolic Alterations (e.g., insulin resistance, visceral adiposity).^[27]^ These biomarkers and physiological measures often co‑exist in individuals with depression and adverse cardiometabolic profiles, and their concurrent presence aligns with patterns of worsened cardiorenal and metabolic status.

Higher levels of multimorbidity commonly co‑occur with depression in CKM populations. Empirical estimates show that participants with depression and two or more chronic conditions have substantially higher observed all‑cause mortality (HR = 2.77; 95% CI: 2.50–3.09) compared with those without this combination.^[28]^

Depression is often accompanied by adverse health behaviors (e.g., poor diet, reduced physical activity, and lower medication adherence)^[29,30]^ and by unfavorable social determinants of health such as low income, lower educational attainment, rural residence, and social isolation.^[31]^ These factors are associated with both higher prevalence of depression and poorer CKM indicators. Representative associations from prior reports include increased odds of advanced CKM with household income below the federal poverty level (OR = 3.68),^[32]^ an association between lower education and higher CKM risk (HR ≈ 1.82) while low educational attainment raises risk by 82% (HR = 1.82),^[33]^ and higher CKM mortality reports from rural versus urban areas (*P* < .01).^[34]^ These observations illustrate overlapping distributions of social disadvantage, adverse behaviors, and biological markers in affected populations.

In our exploratory mediation‑style analysis, several psychosocial and behavioral variables statistically accounted for a notable share of the observed association between PHQ‑9 score and all‑cause mortality. Estimated proportions explained included low income (~15.12%), low physical activity (~10.05%), and marital isolation (~8.96%), with these variables collectively explaining more than one‑third of the association; by contrast, the systemic inflammation index (SIRI) accounted for approximately 12%.^[24]^ These results quantify the relative contribution of measured correlates within the study sample under model assumptions, without implying causal mediation. Across biological, behavioral, and social domains, depression and adverse CKM characteristics frequently co-occur and overlap in ways that correspond to higher observed mortality. The presented findings describe statistical associations and distributions of potential pathway variables rather than causal mechanisms. Integrative approaches that consider biological markers alongside psychosocial and behavioral correlates may therefore be informative for risk stratification and further hypothesis-driven research.

### 4.2. Clinical significance

Given the elevated mortality risk associated with moderate-to-severe depression (PHQ-9 ≥ 10), clinical management should integrate mental health screening with coordinated control of metabolic comorbidities including glycemic and lipid regulation, to mitigate confounding effects. The observed attenuation or reversal of risk at PHQ-9 ≥ 15 warrants cautious interpretation, as it may reflect sample size limitations rather than biological phenomena.

### 4.3. Limitations

As an observational study, causal inference is limited; reverse causation remains possible, for instance, severe illness aggravating depressive symptoms. The use of PHQ-9 ≥ 10 as a clinical threshold may overlook the effects of subclinical depressive symptoms. Additionally, smaller sample size in the highest severity subgroup (PHQ-9 ≥ 15) leads to wider CIs and reduced result stability.

## 5. Conclusion

In this national CKM cohort, depression severity shows a threshold-dependent increase in mortality risk below a PHQ-9 score of 11. Advanced CKM stages drive most deaths, with stronger depression-related risk in younger patients, those with higher CKD risk, or MetS. Protective factors – physical activity, higher income, and marital partnership – explained a significant proportion of the statistical association between depression and mortality.

## Acknowledgments

We extend our sincere gratitude to the NHANES study participants and acknowledge the dedicated staff involved in data collection. During manuscript preparation, the authors utilized AI-assisted tools (Yuanbao, Metaso, Sider, DeepL) for spelling correction, language refinement, and R code optimization. Following tool usage, all content underwent rigorous author review. The author(s) are fully responsible for everything in this publication.

## Author contributions

**Conceptualization:** Yannv Qu.

**Funding acquisition:** Yannv Qu.

**Investigation:** Xiaohong Lin.

**Methodology:** Xiaohong Lin.

**Writing – original draft:** Yannv Qu, Xiaohong Lin.

**Writing – review & editing:** Yannv Qu.

## Supplementary Material


