# Supplementary material for: Nonlinear PHQ-9 thresholds and mortality in cardiovascular-kidney-metabolic syndrome: A prospective cohort study of mediation by socioeconomic factors and physical activity
Source: Medicine (Baltimore). 2026 Jan 2;105(1):e46838. doi: 10.1097/MD.0000000000046838 (PMC12778159; doi:10.1097/MD.0000000000046838)
Supplement: Supplementary file 1 [file medi-105-e46838-s001.pdf]

Supplementary Appendix

Table S1 The basic PREVENT 10-year risk estimation model equations.

**10-year CVD risk assessment equation**	
**Men**	**log-Odds** = -3.031168 + 0.7688528 × (age – 55) /10 + 0.0736174 × ((TC – HDL-C) × 0.02586 – 3.5) – 0.0954431 × (HDL-C × 0.02586 – 1.3) /0.3 – 0.4347345 × (min(SBP, 110) – 110) /20 + 0.3362658 × (max(SBP, 110) – 130) /20 + 0.7692857 × (if diabetes) + 0.4386871 × (if current smoker) + 0.5378979 × (min(eGFR, 60) – 60) / -15 + 0.0164827 × (max(eGFR, 60) – 90) / -15 + 0.288879 × (if using anti hypertensive medication) – 0.1337349 × (if using statin) – 0.0475924 × (if using anti-hypertensive medication) × (max(SBP, 110) – 130) /20 + 0.150273 × (if using statin) × ((TC – HDL-C) × 0.02586 – 3.5) – 0.0517874 × (age – 55) /10 × ((TC – HDL-C) × 0.02586 – 3.5) + 0.0191169 × (age – 55) /10 × (HDL-C × 0.02586 – 1.3) /0.3 – 0.1049477 × (age – 55) /10 × (max(SBP, 110) – 130) /20 – 0.2251948 × (age – 55) /10 × (if diabetes) – 0.0895067 × (age – 55) /10 × (if current smoker) – 0.1543702 × (age – 55) /10 × (min(eGFR, 60) – 60) / -15
	**Risk**= 1 / (1 + exp(-log-Odds))
**Women**	log-Odds = -3.307728 + 0.7939329 × (age – 55) /10 + 0.0305239 × ((TC – HDL-C) × 0.02586 – 3.5) – 0.1606857 × (HDL-C × 0.02586 – 1.3) /0.3 – 0.2394003 × (min(SBP, 110) – 110) /20 + 0.360078 × (max(SBP, 110) – 130) /20 + 0.8667604 × (if diabetes) + 0.5360739 × (if current smoker) + 0.6045917 × (min(eGFR, 60) – 60) / -15 + 0.0433769 × (max(eGFR, 60) – 90) / -15 + 0.3151672 × (if using anti hypertensive medication) – 0.1477655 × (if using statin) – 0.0663612 × (if using anti-hypertensive medication) × (max(SBP, 110) – 130) /20 + 0.1197879 × (if using statin) × ((TC – HDL-C) × 0.02586 – 3.5) – 0.0819715 × (age – 55) /10 × ((TC – HDL-C) × 0.02586 – 3.5) + 0.0306769 × (age – 55) /10 × (HDL-C × 0.02586 × 0.02586 – 1.3) /0.3 – 0.0946348 × (age – 55) /10 × (max(SBP, 110) – 130) /20 – 0.27057 × (age – 55) /10 × (if diabetes) – 0.078715 × (age – 55) /10 × (if current smoker) – 0.1637806 × (age – 55) /10 × (min(eGFR, 60) – 60) / -15
	**Risk**= 1 / (1 + exp(-log-Odds))

Abbreviations: TC, total cholesterol; HDL-C, high-density lipoprotein cholesterol; SBP, systolic blood pressure; eGFR, estimated glomerular filtration rate.

Table S2 Definition of CKM syndrome staging adjusted for data in NHANES.

CKM syndrome stages	Definition
Stage 0: No CKM health risk factors	Individuals without overweight/obesity, metabolic risk factors (hypertriglyceridemia, hypertension, diabetes, MeTS), CKD or subclinical/clinical CVD<br>(1)BMI between 18.5 and 25 kg/m^2, inclusive<br>(2)WC <102 cm for men or <88 cm for women
Stage 1: Excess or dysfunctional adiposity	Individuals with overweight/obesity, abdominal obesity, or adipose tissue dysfunction without other metabolic risk factors, CKD, or subclinical/clinical CVD<br>(1)BMI ≥ 25 kg/m^2<br>(2)WC ≥ 102 cm for men or ≥ 88 cm for women<br>(3)FBG levels ranging from 100 to 124 mg/dL, or HbA1c levels between 5.7 and 6.4 %
Stage 2: Metabolic risk factors and moderate to high-risk CKD	Individuals with metabolic risk factors (hypertriglyceridemia, hypertension, diabetes, MeTS*) or moderate to high-risk CKD stage(The stage of CKD is determined by the KDIGO criteria, using eGFR and UACR. The eGFR was calculated using the 2021 race and ethnicity- free Chronic Kidney Disease Epidemiology Collaboration creatinine equation.<br>(1)TG >135 mg/dL<br>(2)Hypertension is defined by an SBP of ≥130 mm Hg, a DBP of ≥80 mm Hg, a medical diagnosis, or taking antihypertensive medication.<br>(3)Diabetes is defined by FBG levels of > 126 mg/dL, HbA1c levels of ≥ 6.5%, a medical diagnosis, or taking insulin or glucose-lowering medication.<br>(4) Moderate to high-risk CKD in the KDIGO classification is defined as UACR ≥ 30 mg/g and eGFR ≥ 60 ml/min/1.73m^2, UACR < 300 mg/g and eGFR ≤ 45-59 ml/min/1.73m^2, or UACR < 30 mg/g and eGFR ≤ 30-44 ml/min/1.73m^2.
Stage 3: Subclinical CVD in CKM	Risk equivalents for subclinical CVD: high predicted 10-year CVD risk or very high-risk KDIGO CKD stage<br>(1)A high 10-year CVD risk is defined as a 20% or above risk, as determined by the basic Predicting Risk of CVD EVENTs (PREVENT) equation.<br>(2)Very high-risk CKD in the KDIGO classification is defined as UACR ≥ 300 mg/g and eGFR ≤ 45-59 ml/min/1.73 m^2, UACR ≥ 30 mg/g and eGFR ≤ 30-44 ml/min/1.73 m^2, or eGFR ≤ 29 ml/min/1.73 m^2.
Stage 4: Clinical CVD in CKM	Clinical CVD (self-reported diagnosed cardiovascular disease, including heart failure, coronary heart disease, angina, heart attack, and stroke) in individuals

Abbreviations: CKM syndrome, Cardiovascular-Kidney-Metabolic syndrome; NHANES, National Health and Nutrition Examination Survey; MeTS, metabolic syndrome; CKD, chronic kidney disease; CVD, cardiovascular disease; BMI, body mass index; WC, waist circumference; FBG, fasting blood glucose; HbA1c, glycated hemoglobin A1c; KDIGO, Kidney Disease Improving Global Outcomes; UACR, urine albumin-to-creatinine ratio; eGFR, estimated glomerular filtration rate; TG, triglycerides; SBP, systolic blood pressure; DBP, diastolic blood pressure.

* MeTS is defined by the presence of ≥3 of the following: (1) WC ≥ 102 cm for men or ≥ 88 cm for women; (2) HDL-C <40 mg/dL for men, <50 mg/dL for women; (3) TG ≥150 mg/dL; (4) Elevated blood pressure (SBP ≥130 mm Hg, DBP ≥80 mm Hg, a medical diagnosis, or taking antihypertensive medication); (5) FBG ≥100 mg/dL

Table S3 Variables, Measurement Methods, and Equipment/Techniques.

Variable	Measurement Method	Equipment/Technique
Body Mass Index (BMI)	Calculated from measured height and weight: BMI = weight (kg) / $height^2$ (m^2)	Calibrated scales, stadiometers
Systolic/Diastolic Blood Pressure (SBP/DBP)	Mercury sphygmomanometer in seated position, with three measurements averaged for accuracy.	Mercury sphygmomanometer
Serum Creatinine	Enzymatic method	Roche/Hitachi Modular P analyzer
High-Density Lipoprotein Cholesterol (HDL-C)	Direct enzymatic colorimetric method	Roche/Hitachi Modular P analyzer
Total Cholesterol	Enzymatic method	Roche/Hitachi Modular P analyzer
Glycohemoglobin	High-Performance Liquid Chromatography (HPLC)	Tosoh Automated Glycohemoglobin Analyzer
Albumin-Creatinine Ratio (ACR)	Urine assay	Immunoassay for albumin, enzymatic assay for creatinine
Complete blood count	A single beam photometer for hemoglobinometry. The WBC differential uses VCS (volume, conductivity and scatter) technology	The Beckman Coulter DxH 800 instrument

Table S4. Baseline characteristics of patients with CKM syndrome stages 0–4 concerning all-cause mortality and cardiovascular mortality (N = 27,673).

		All-cause mortality			Cardiovascular mortality		
		Survivors	Deceased	P-value	Survivors	Deceased	P-value
TC, mg/dL	192.7 ± 41.8	193.1 ± 41.1	188.2 ± 44.6	<0.001	192.9 ± 41.6	186.8 ± 45.3	0.004
LDL-C, mg/dL	113.6 ± 35.4	114.3 ± 35.1	106.3 ± 37.7	<0.001	113.8 ± 35.3	105.8 ± 39.0	<0.001
HDL-C, mg/dL	52.5 ± 15.9	52.6 ± 15.9	52.7 ± 17.7	0.919	52.7 ± 16.1	51.9 ± 16.4	0.244
TG, mg/dL	155.5 ± 134.4	155.3 ± 136.5	158.0 ± 109.6	0.358	155.6 ± 135.2	155.1 ± 96.7	0.922
Standing height, cm	167.0 ± 10.2	167.2 ± 10.2	165.8 ± 10.4	<0.001	167.1 ± 10.2	165.2 ± 10.2	<0.001
Albumin, g/L	42.6 ± 3.4	42.8 ± 3.3	41.1 ± 3.4	<0.001	42.7 ± 3.4	41.1 ± 3.4	<0.001
Creatinine, mg/dL	0.9 ± 0.3	0.9 ± 0.3	1.1 ± 0.4	<0.001	0.9 ± 0.4	1.2 ± 0.9	<0.001
ALP, U/L	68.7 ± 23.3	68.1 ± 22.8	75.3 ± 27.4	<0.001	68.6 ± 23.1	75.2 ± 28.1	<0.001
Total calcium, mg/dL	9.4 ± 0.4	9.4 ± 0.4	9.4 ± 0.4	0.866	9.4 ± 0.4	9.4 ± 0.4	0.546
UA, mg/dL	5.5 ± 1.4	5.4 ± 1.4	6.0 ± 1.6	<0.001	5.4 ± 1.4	6.1 ± 1.7	<0.001
BUN, mg/dL	13.4 ± 5.7	13.0 ± 5.1	17.7 ± 9.2	<0.001	13.3 ± 5.5	19.0 ± 9.3	<0.001
UACR, mg/g	36.6 ± 634.2	31.9 ± 646.7	86.8 ± 479.1	<0.001	35.1 ± 637.3	95.6 ± 496.6	0.013
serum 25(OH)D levels, nmol/L	63.8 ± 27.3	63.6 ± 27.0	66.3 ± 30.0	<0.001	63.7 ± 27.1	67.3 ± 31.7	<0.001
eGFR, ml/min/1.73m2	95.6 ± 22.7	97.8 ± 21.4	73.1 ± 23.7	<0.001	96.4 ± 22.2	69.2 ± 22.8	<0.001
Stroke, n (%)	962 (3.5%)	644 (2.6%)	318 (12.9%)	<0.001	853 (3.2%)	109 (14.6%)	<0.001
Cancer, n (%)	2470 (8.9%)	1891 (7.5%)	579 (23.5%)	<0.001	2320 (8.6%)	150 (20.1%)	<0.001

Abbreviations: BMI,body mass index;SBP, systolic blood pressure; DBP, diastolic blood pressure; CI,confidence interval; HR, hazard ratio; DM, diabetes mellitus; eGFR; estimated glomerular filtration rate;FBG, fasting blood glucose;HbA1c, hemoglobin A1c; HDL-C, high-density lipoprotein cholesterol; LDL-C, low-density lipoprotein cholesterol; TC, total cholesterol; TG, triglyceride; UACR, urinary albumin creatinine ratio ; ALP, alkaline phosphotase; UA, uric acid; BUN, blood urea nitrogen; CKD , Chronic kidney disease ; MeTS, metabolic syndrome; CKM, Cardiovascular-Kidney-Metabolic Syndrome. Frequencies are expressed as absolute numbers and percentages (%); values are means (standard deviation). Among the 23,635 patients,the amount of missing values for the covariates were 12123 (51.3%) for education level, 9134 (38.6%) for marital status,10911 (46.2%)for poverty income ratio, 349 (<0.1%) for smoking, 8885 (37.1%) for drinking,11385 (48.2%) for FBS, 11049 (46.7%) for LDL-C. Numbers not totalling 100% are due to missing data. Dummy variables were used to indicate missing covariate values.

Table S5. Baseline characteristics of patients with CKM syndrome stages 0–4 concerning all-cause mortality and cardiovascular mortality, weighted for representativeness (N = 27,673).

		All-cause mortality			Cardiovascular mortality		
		Survivors	Deceased	P-value	Survivors	Deceased	P-value
Age, years	46.73 (46.18 ,47.27)	45.30 (44.75 ,45.85)	68.49 (67.35 ,69.62)	<0.0001	46.27 (45.72 ,46.82)	70.76 (69.17 ,72.35)	<0.0001
Gender, n (%)				0.0016			0.0003
Male	48.38 (47.44 ,49.32)	48.05 (47.06 ,49.04)	53.40 (50.24 ,56.53)		48.17 (47.21 ,49.14)	59.16 (53.18 ,64.88)	
Female	51.62 (50.68 ,52.56)	51.95 (50.96 ,52.94)	46.60 (43.47 ,49.76)		51.83 (50.86 ,52.79)	40.84 (35.12 ,46.82)	
Race, n (%)				<0.0001			<0.0001
Mexican American	8.64 (7.13 ,10.43)	8.96 (7.41 ,10.80)	3.71 (2.44 ,5.60)		8.73 (7.21 ,10.54)	3.92 (2.26 ,6.70)	
Other Hispanic	5.90 (4.80 ,7.23)	6.12 (4.98 ,7.51)	2.49 (1.63 ,3.80)		5.97 (4.86 ,7.31)	2.06 (0.90 ,4.62)	
Non-Hispanic White	66.67 (63.35 ,69.83)	65.87 (62.52 ,69.07)	78.84 (74.13 ,82.89)		66.47 (63.14 ,69.64)	77.24 (70.89 ,82.56)	
Non-Hispanic Black	11.40 (9.76 ,13.26)	11.42 (9.79 ,13.29)	11.02 (8.33 ,14.44)		11.37 (9.74 ,13.23)	12.90 (9.20 ,17.81)	
Other Race - Including Multi-Racial	4.17 (3.51 ,4.94)	4.25 (3.57 ,5.05)	2.90 (1.84 ,4.53)		4.19 (3.53 ,4.96)	2.90 (1.35 ,6.10)	
Non-Hispanic Asian	3.23 (2.66 ,3.93)	3.38 (2.78 ,4.10)	1.04 (0.59 ,1.82)		3.28 (2.70 ,3.97)	0.98 (0.37 ,2.60)	
Education Level, n (%)				<0.001			<0.001
Less Than High School Grad	16.86 (15.37 ,18.47)	16.04 (14.49 ,17.71)	29.41 (25.59 ,33.56)		16.67 (15.16 ,18.29)	27.06 (21.79 ,33.06)	
High School Grad/GED or Equivalent	21.53 (20.01 ,23.14)	21.08 (19.51 ,22.74)	28.48 (25.48 ,31.68)		21.36 (19.82 ,22.98)	30.59 (24.62 ,37.29)	
Some College or above	60.08 (57.63 ,62.48)	61.28 (58.75 ,63.74)	41.83 (37.46 ,46.34)		60.42 (57.93 ,62.85)	42.35 (36.54 ,48.38)	
Marital Status, n (%)				<0.0001			0.0001
Married or Living with partner	63.38 (61.79 ,64.94)	63.95 (62.33 ,65.55)	54.69 (51.26 ,58.07)		63.60 (61.98 ,65.19)	51.76 (45.51 ,57.96)	
Separated or Never married	35.08 (33.53 ,36.67)	34.42 (32.84 ,36.05)	45.14 (41.76 ,48.57)		34.83 (33.26 ,36.45)	48.24 (42.04 ,54.49)	
Poverty income ratio, n (%)				<0.001			<0.001
≤1.30	24.48 (22.66 ,26.39)	24.17 (22.31 ,26.14)	29.17 (25.38 ,33.27)		24.49 (22.65 ,26.43)	23.79 (19.00 ,29.35)	

1.3~1.85	12.03 (11.23 ,12.88)	11.62 (10.77 ,12.54)	18.24 (16.24 ,20.43)		11.85 (11.03 ,12.72)	21.63 (16.59 ,27.68)	
> 1.85	58.75 (56.42 ,61.04)	59.51 (57.13 ,61.84)	47.19 (43.14 ,51.28)		58.92 (56.56 ,61.24)	49.79 (43.44 ,56.15)	
Smoking, n (%)				<0.0001			0.0055
Never	55.32 (53.68 ,56.95)	56.33 (54.67 ,57.97)	39.97 (36.24 ,43.81)		55.45 (53.79 ,57.10)	48.42 (41.84 ,55.06)	
Current smoker	19.14 (17.85 ,20.49)	19.06 (17.72 ,20.47)	20.42 (17.38 ,23.84)		19.20 (17.88 ,20.60)	15.75 (10.46 ,23.03)	
Ever smoker	24.49 (23.05 ,25.99)	23.52 (21.99 ,25.13)	39.19 (35.11 ,43.42)		24.27 (22.80 ,25.81)	35.83 (28.89 ,43.42)	
Drinking, g/year	26.98 (20.16 ,33.81)	24.38 (19.46 ,29.31)	66.58 (-24.34 ,157.51)	0.3698	27.17 (20.22 ,34.11)	17.40 (2.25 ,32.56)	0.2521
FBS, mg/dL	105.85 (105.09 ,106.60)	104.90 (104.18 ,105.62)	120.35 (116.52 ,124.18)	<0.0001	105.58 (104.84 ,106.33)	119.68 (113.86 ,125.49)	<0.0001
HBA1c, %	5.63 (5.60 ,5.65)	5.59 (5.57 ,5.62)	6.11 (6.01 ,6.22)	<0.0001	5.62 (5.59 ,5.64)	6.21 (6.01 ,6.42)	<0.0001
eGFR, ml/min/1.73m2	97.78 (97.15 ,98.40)	99.24 (98.56 ,99.91)	75.41 (73.32 ,77.49)	<0.0001	98.27 (97.65 ,98.90)	71.55 (68.14 ,74.96)	<0.0001
Waist circumference, cm	99.24 (98.71 ,99.78)	99.03 (98.47 ,99.58)	102.74 (101.64 ,103.84)	<0.0001	99.16 (98.62 ,99.70)	104.03 (102.05 ,106.02)	<0.0001
BMI,kg/m^2	29.05 (28.85 ,29.26)	29.07 (28.85 ,29.28)	28.89 (28.43 ,29.35)	0.4865	29.05 (28.84 ,29.25)	29.51 (28.74 ,30.28)	0.2448
PHQ9 Score	2.78 (2.67 ,2.88)	2.76 (2.65 ,2.87)	3.03 (2.66 ,3.41)	0.1657	2.77 (2.66 ,2.88)	3.00 (2.46 ,3.53)	0.4258
Physical activity, n (%)	57.98 (56.54 ,59.41)	59.59 (58.17 ,61.00)	33.47 (29.46 ,37.74)		58.50 (57.04 ,59.95)	30.62 (23.96 ,38.20)	<0.001
Diabetes, n (%)	9.29 (8.55 ,10.08)	8.11 (7.44 ,8.84)	27.25 (24.09 ,30.66)		8.93 (8.21 ,9.70)	28.47 (22.64 ,35.14)	<0.001
Hypertension, n (%)	32.38 (31.01 ,33.78)	30.34 (28.94 ,31.77)	63.53 (59.18 ,67.66)		31.73 (30.36 ,33.14)	66.49 (59.07 ,73.17)	<0.001
Cardiovascular disease, n (%)	6.78 (6.12 ,7.49)	5.32 (4.75 ,5.96)	28.99 (24.99 ,33.34)		6.21 (5.59 ,6.90)	36.46 (30.63 ,42.72)	<0.001
Hyperlipidemia,n (%)	31.89 (30.67 ,33.14)	30.89 (29.59 ,32.23)	47.14 (43.23 ,51.09)		31.43 (30.20 ,32.70)	56.05 (50.17 ,61.77)	<0.001
Antihypertensive agents,n (%)	27.50 (26.19 ,28.84)	25.31 (24.00 ,26.67)	60.85 (56.32 ,65.19)		26.78 (25.47 ,28.12)	65.37 (57.98 ,72.10)	<0.001
Antihyperlipidemic agents,n (%)	22.46 (21.36 ,23.60)	21.10 (19.96 ,22.28)	43.25 (39.80 ,46.77)		21.89 (20.80 ,23.01)	52.56 (45.98 ,59.06)	<0.001
Antihyperglycemic agents,n (%)	77.52 (74.00 ,80.68)	78.65 (75.13 ,81.80)	72.38 (64.94 ,78.75)		77.51 (74.09 ,80.60)	77.61 (64.90 ,86.67)	0.9842
CKD Risk, n (%)				<0.001			<0.001

Low-risk	89.70 (88.91 ,90.44)	91.23 (90.46 ,91.95)	66.34 (62.59 ,69.90)		90.29 (89.51 ,91.01)	59.05 (50.71 ,66.90)	
Moderate to high-risk	9.35 (8.61 ,10.15)	8.22 (7.52 ,8.97)	26.67 (23.04 ,30.66)		8.87 (8.16 ,9.64)	34.38 (27.06 ,42.53)	
Very high-risk	0.95 (0.81 ,1.11)	0.55 (0.45 ,0.67)	6.98 (5.26 ,9.22)		0.84 (0.72 ,0.98)	6.57 (3.58 ,11.74)	
MetS, n (%)	38.33 (36.90 ,39.79)	37.17 (35.70 ,38.68)	55.99 (52.22 ,59.69)		37.96 (36.51 ,39.43)	57.95 (52.37 ,63.34)	<0.001
CKM_Stage, n (%)				<0.001			<0.001
Stage 0	21.40 (20.15 ,22.70)	22.33 (21.03 ,23.69)	7.13 (5.48 ,9.24)		21.74 (20.47 ,23.06)	3.42 (1.72 ,6.68)	
Stage 1	33.13 (31.98 ,34.29)	34.63 (33.45 ,35.83)	10.20 (7.94 ,13.02)		33.60 (32.46 ,34.77)	8.15 (5.33 ,12.28)	
Stage 2	35.30 (33.99 ,36.63)	36.15 (34.77 ,37.55)	22.39 (19.04 ,26.12)		35.63 (34.29 ,36.99)	18.00 (14.29 ,22.43)	
Stage 3	2.86 (2.55 ,3.20)	0.15 (0.10 ,0.23)	44.17 (40.19 ,48.23)		1.91 (1.66 ,2.19)	52.79 (45.97 ,59.51)	
Stage 4	7.32 (6.67 ,8.03)	6.74 (6.14 ,7.40)	16.11 (13.07 ,19.70)		7.12 (6.47 ,7.83)	17.63 (12.50 ,24.29)	

Continuous data were expressed as weighted means with 95% confidence intervals (CIs), and the corresponding P values were derived from survey-weighted linear regression analyses. In contrast, categorical data were represented as weighted proportions (95% CI), with P values calculated using the survey-weighted Chi-square test.

Abbreviations: BMI,body mass index;SBP, systolic blood pressure; DBP, diastolic blood pressure; CI,confidence interval; HR, hazard ratio; DM, diabetes mellitus; eGFR; estimated glomerular filtration rate;FBG, fasting blood glucose;HbA1c, hemoglobin A1c; HDL-C, high-density lipoprotein cholesterol; LDL-C, low-density lipoprotein cholesterol; TC, total cholesterol; TG, triglyceride; UACR, urinary albumin creatinine ratio ; ALP, alkaline phosphotase; UA, uric acid; BUN, blood urea nitrogen; CKD , Chronic kidney disease ; MeTS, metabolic syndrome; CKM, Cardiovascular-Kidney-Metabolic Syndrome. Frequencies are expressed as absolute numbers and percentages (%); values are means (standard deviation). Among the 23,635 patients,the amount of missing values for the covariates were 12123 (51.3%) for education level, 9134 (38.6%) for marital status,10911 (46.2%)for poverty income ratio, 349 (<0.1%) for smoking, 8885 (37.1%) for drinking,11385 (48.2%) for FBS, 11049 (46.7%) for LDL-C. Numbers not totalling 100% are due to missing data. Dummy variables were used to indicate missing covariate values.

Table S6. Comparison of characteristics between participants with and without depression (N = 27,673).

	Depression		
	No	Yes	p-value
N	25390	2283	
Age, years	48.5 ± 18.1	48.2 ± 16.4	0.478
Gender, n (%)			<0.001
Male	12617 (49.7%)	813 (35.6%)	
Female	12773 (50.3%)	1470 (64.4%)	
Race, n (%)			<0.001
Mexican American	4000 (15.8%)	359 (15.7%)	
Other Hispanic	2676 (10.5%)	342 (15.0%)	
Non-Hispanic White	10379 (40.9%)	947 (41.5%)	
Non-Hispanic Black	5427 (21.4%)	488 (21.4%)	
Other Race - Including Multi-Racial	979 (3.9%)	98 (4.3%)	
Non-Hispanic Asian	1929 (7.6%)	49 (2.1%)	
Education Level, n (%)			<0.001
Less Than High School Grad	6067 (23.9%)	842 (36.9%)	
High School Grad/GED or Equivalent	5580 (22.0%)	525 (23.0%)	
Some College or above	13093 (51.6%)	866 (37.9%)	
Marital Status, n (%)			<0.001
Married or Living with partner	15071 (59.4%)	998 (43.7%)	
Separated or Never married	9682 (38.1%)	1236 (54.1%)	
Poverty income ratio, n (%)			<0.001
≤1.30	8323 (32.8%)	1223 (53.6%)	
1.3~1.85	3459 (13.6%)	315 (13.8%)	
> 1.85	11981 (47.2%)	598 (26.2%)	
Smoking, n (%)			<0.001
Never	14364 (56.6%)	923 (40.4%)	
Current smoker	4731 (18.6%)	850 (37.2%)	
Ever smoker	5878 (23.2%)	479 (21.0%)	
Drinking, g/year	38.9 ± 400.7	58.8 ± 392.8	0.080
Albumin, g/L	42.7 ± 3.3	41.8 ± 3.5	<0.001

Creatinine, mg/dL	0.9 ± 0.4	0.9 ± 0.6	0.570
FBS, mg/dL	108.8 ± 35.8	114.3 ± 44.0	<0.001
HBA1c, %	5.7 ± 1.1	5.9 ± 1.3	0.478
TC, mg/dL	192.4 ± 41.5	195.9 ± 44.5	<0.001
LDL-C, mg/dL	113.4 ± 35.2	115.7 ± 37.1	0.055
HDL-C, mg/dL	52.8 ± 16.1	50.9 ± 16.0	<0.001
TG, mg/dL	154.1 ± 132.6	171.7 ± 151.4	<0.001
BUN, mg/dL	13.5 ± 5.7	12.8 ± 6.3	<0.001
UACR, mg/g	35.0 ± 653.0	54.2 ± 367.9	0.168
eGFR, ml/min/1.73m2	95.7 ± 22.6	95.2 ± 23.5	0.371
Standing height, cm	167.2 ± 10.2	165.2 ± 9.9	<0.001
Waist circumference, cm	98.9 ± 15.9	102.9 ± 18.0	<0.001
BMI,kg/m²	29.0 ± 6.7	31.1 ± 8.3	<0.001
PHQ9 Score	1.9 ± 2.4	14.3 ± 3.9	<0.001
Physical activity, n (%)	13603 (53.6%)	886 (38.8%)	<0.001
Diabetes, n (%)	2998 (11.8%)	444 (19.4%)	<0.001
Hypertension, n (%)	12617 (49.7%)	813 (35.6%)	<0.001
Cardiovascular disease, n (%)	1841 (7.3%)	325 (14.2%)	<0.001
Stroke, n (%)	801 (3.2%)	161 (7.1%)	<0.001
Cancer, n (%)	2228 (8.8%)	242 (10.6%)	0.003
Hyperlipidemia,n (%)	7996 (31.5%)	918 (40.2%)	<0.001
Antihypertensive agents,n (%)	10379 (40.9%)	947 (41.5%)	<0.001
Antihyperlipidemic agents,n (%)	5427 (21.4%)	488 (21.4%)	<0.001
Antihyperglycemic agents,n (%)	979 (3.9%)	98 (4.3%)	<0.001
CKD Risk, n (%)			<0.001
Low-risk	22247 (87.6%)	1915 (83.9%)	
Moderate to high-risk	2798 (11.0%)	320 (14.0%)	
Very high-risk	345 (1.4%)	48 (2.1%)	
MetS, n (%)	9147 (36.0%)	1072 (47.0%)	<0.001
CKM_Stage, n (%)			<0.001
Stage 0	5216 (20.5%)	344 (15.1%)	
Stage 1	8705 (34.3%)	619 (27.1%)	

Stage 2	8445 (33.3%)	854 (37.4%)	
Stage 3	1086 (4.3%)	86 (3.8%)	
Stage 4	1938 (7.6%)	380 (16.6%)	
Cardiovascular mortality	673 (2.7%)	72 (3.2%)	0.155
All-cause mortality	2231 (8.8%)	237 (10.4%)	0.010

Frequencies are expressed as absolute numbers and percentages (%); values are means (standard deviation).

Abbreviations: HR, hazard ratio; CI, confidence interval; BMI, body mass index; SBP, systolic blood pressure; DBP, diastolic blood pressure; DM, diabetes mellitus; eGFR, estimated glomerular filtration rate; FBG, fasting blood glucose; HbA1c, hemoglobin A1c; HDL-C, high-density lipoprotein cholesterol; LDL-C, low-density lipoprotein cholesterol; TC, total cholesterol; TG, triglyceride; UACR, urinary albumin creatinine ratio； BUN, blood urea nitrogen; CKD, Chronic kidney disease； MeTS, metabolic syndrome; CKM, Cardiovascular-Kidney-Metabolic Syndrome.

Table S7. Comparison of Clinical and Metabolic Characteristics Between Participants With CKM syndrome stage 0–4 (N = 27,673).

CKM_Stage	Stage 0	Stage 1	Stage 2	Stage 3	Stage 4	P-value
	5560	9324	9299	1172	2318	
Age, years	39.7 ± 16.6	43.2 ± 15.9	51.7 ± 15.9	76.5 ± 7.1	63.3 ± 12.8	<0.001
**Gender**, n (%)						<0.001
Male	2867 (51.6%)	4383 (47.0%)	4227 (45.5%)	679 (57.9%)	1274 (55.0%)	
Female	2693 (48.4%)	4941 (53.0%)	5072 (54.5%)	493 (42.1%)	1044 (45.0%)	
**Race**, n (%)						<0.001
Mexican American	633 (11.4%)	1625 (17.4%)	1761 (18.9%)	88 (7.5%)	252 (10.9%)	
Other Hispanic	579 (10.4%)	1091 (11.7%)	1058 (11.4%)	58 (4.9%)	232 (10.0%)	
Non-Hispanic White	2344 (42.2%)	3390 (36.4%)	3712 (39.9%)	786 (67.1%)	1094 (47.2%)	
Non-Hispanic Black	1075 (19.3%)	2325 (24.9%)	1767 (19.0%)	184 (15.7%)	564 (24.3%)	
Other Race - Including Multi-Racial	309 (5.6%)	330 (3.5%)	313 (3.4%)	36 (3.1%)	89 (3.8%)	
Non-Hispanic Asian	620 (11.2%)	563 (6.0%)	688 (7.4%)	20 (1.7%)	87 (3.8%)	
**Education Level**, n (%)						<0.001
Less Than High School Grad	1025 (18.4%)	2131 (22.9%)	2540 (27.3%)	439 (37.5%)	774 (33.4%)	
High School Grad/GED or Equivalent	1108 (19.9%)	1946 (20.9%)	2178 (23.4%)	294 (25.1%)	579 (25.0%)	
Some College or above	3091 (55.6%)	4999 (53.6%)	4471 (48.1%)	437 (37.3%)	961 (41.5%)	
**Marital Status**, n (%)			<0.001			<0.001
Married or Living with partner	14846 (58.9%)	1223 (49.6%)		15716 (58.4%)	353 (47.4%)	
Separated or Never married	9677 (38.4%)	1241 (50.3%)		10526 (39.1%)	392 (52.6%)	
**Poverty income ratio**, n (%)						<0.001

≤1.30	1839 (33.1%)	3090 (33.1%)	3321 (35.7%)	370 (31.6%)	926 (39.9%)	
1.3~1.85	690 (12.4%)	1220 (13.1%)	1262 (13.6%)	231 (19.7%)	371 (16.0%)	
> 1.85	2639 (47.5%)	4426 (47.5%)	4150 (44.6%)	497 (42.4%)	867 (37.4%)	
**Smoking, n (%)**						<0.001
Never	3114 (56.0%)	5606 (60.1%)	5140 (55.3%)	501 (42.7%)	926 (39.9%)	
Current smoker	1354 (24.4%)	1719 (18.4%)	1819 (19.6%)	129 (11.0%)	560 (24.2%)	
Ever smoker	871 (15.7%)	1840 (19.7%)	2273 (24.4%)	542 (46.2%)	831 (35.8%)	
Drinking, g/year	36.4 ± 229.8	35.3 ± 221.6	48.0 ± 596.2	26.5 ± 156.6	49.6 ± 373.2	0.368
Creatinine, mg/dL	0.8 ± 0.2	0.8 ± 0.2	0.9 ± 0.4	1.4 ± 1.1	1.0 ± 0.6	<0.001
FBS, mg/dL	96.6 ± 18.5	98.5 ± 19.8	120.3 ± 45.1	121.1 ± 36.6	123.9 ± 49.5	<0.001
HBA1c, %	5.4 ± 0.6	5.5 ± 0.7	6.1 ± 1.3	6.2 ± 1.1	6.3 ± 1.4	<0.001
TC, mg/dL	184.6 ± 38.4	193.9 ± 39.1	200.0 ± 43.4	182.0 ± 42.9	182.3 ± 44.9	<0.001
LDL-C, mg/dL	107.2 ± 33.0	117.0 ± 33.2	118.5 ± 36.7	101.5 ± 35.0	102.4 ± 37.1	<0.001
HDL-C, mg/dL	60.3 ± 16.5	55.7 ± 14.6	46.3 ± 14.3	51.9 ± 15.9	50.1 ± 16.6	<0.001
TG, mg/dL	102.7 ± 68.0	120.9 ± 85.8	213.4 ± 173.3	154.2 ± 100.0	169.0 ± 137.6	<0.001
BUN, mg/dL	12.1 ± 3.9	12.3 ± 4.1	13.6 ± 5.5	22.0 ± 10.6	15.8 ± 7.3	<0.001
UACR, mg/g	24.6 ± 1253.4	7.2 ± 37.7	47.2 ± 315.3	172.8 ± 967.7	74.8 ± 393.8	<0.001
eGFR, ml/min/1.73m2	104.8 ± 17.6	101.7 ± 18.1	93.5 ± 22.1	59.1 ± 21.7	79.3 ± 22.5	<0.001
Standing height, cm	168.2 ± 10.0	167.2 ± 10.0	166.6 ± 10.5	164.7 ± 10.2	166.7 ± 10.1	<0.001
Waist circumference, cm	81.2 ± 6.6	99.9 ± 12.3	107.1 ± 15.3	102.0 ± 14.7	106.2 ± 16.4	<0.001
BMI,kg/m²	22.2 ± 1.7	30.2 ± 5.6	32.1 ± 6.9	28.2 ± 5.9	31.0 ± 7.5	<0.001
PHQ9 Score	2.5 ± 3.8	2.6 ± 3.9	3.2 ± 4.4	2.8 ± 4.0	4.5 ± 5.6	<0.001

Physical activity, n (%)	3449 (62.0%)	5377 (57.7%)	4475 (48.1%)	308 (26.3%)	880 (38.0%)	<0.001
Diabetes, n (%)	115 (2.1%)	375 (4.0%)	1839 (19.8%)	368 (31.4%)	745 (32.1%)	<0.001
Hypertension, n (%)	673 (12.1%)	1751 (18.8%)	4719 (50.7%)	882 (75.3%)	1678 (72.4%)	<0.001
Cardiovascular disease, n (%)	0 (0.0%)	0 (0.0%)	0 (0.0%)	370 (31.6%)	1796 (77.5%)	<0.001
Stroke, n (%)	0 (0.0%)	0 (0.0%)	0 (0.0%)	181 (15.4%)	781 (33.7%)	<0.001
Cancer, n (%)	314 (5.6%)	539 (5.8%)	881 (9.5%)	319 (27.2%)	417 (18.0%)	<0.001
Hyperlipidemia,n (%)	846 (15.2%)	2217 (23.8%)	3867 (41.6%)	562 (48.0%)	1422 (61.3%)	<0.001
Antihypertensive agents,n (%)	477 (8.6%)	1407 (15.1%)	4090 (44.0%)	861 (73.5%)	1592 (68.7%)	<0.001
Antihyperlipidemic agents,n (%)	409 (7.4%)	1222 (13.1%)	2912 (31.3%)	521 (44.5%)	1381 (59.6%)	<0.001
Antihyperglycemic agents,n (%)	76 (66.1%)	187 (49.9%)	1418 (77.1%)	247 (67.1%)	572 (76.8%)	<0.001
CKD Risk, n (%)			<0.001			<0.001
Low-risk	5560 (100.0%)	9324 (100.0%)	7054 (75.9%)	538 (45.9%)	1686 (72.7%)	
Moderate to high-risk	0 (0.0%)	0 (0.0%)	2157 (23.2%)	432 (36.9%)	529 (22.8%)	
Very high-risk	0 (0.0%)	0 (0.0%)	88 (0.9%)	202 (17.2%)	103 (4.4%)	
MetS, n (%)	0 (0.0%)	0 (0.0%)	8312 (89.4%)	607 (51.8%)	1300 (56.1%)	<0.001
All-cause mortality	183 (3.3%)	327 (3.5%)	463 (5.0%)	1115 (95.1%)	380 (16.4%)	<0.001
Cardiovascular mortality	26 (0.5%)	81 (0.9%)	112 (1.2%)	401 (34.2%)	125 (5.4%)	<0.001

Frequencies are expressed as absolute numbers and percentages (%); values are means (standard deviation).

Abbreviations: HR, hazard ratio; CI, confidence interval; BMI, body mass index; SBP, systolic blood pressure; DBP, diastolic blood pressure; DM, diabetes mellitus; eGFR, estimated glomerular filtration rate; FBG, fasting blood glucose; HbA1c, hemoglobin A1c; HDL-C, high-density lipoprotein cholesterol; LDL-C, low-density lipoprotein cholesterol; TC, total cholesterol; TG, triglyceride; UACR, urinary albumin creatinine ratio；BUN, blood urea nitrogen; CKD, Chronic kidney disease；MeTS, metabolic syndrome; CKM, Cardiovascular-Kidney-Metabolic Syndrome.

Cardiovascular-Kidney-Metabolic Syndrome. Frequencies are expressed as absolute numbers and percentages (%); values are means (standard deviation). Among the 23,635 patients,the amount of missing values for the covariates were 12123 (51.3%) for education level, 9134 (38.6%) for marital status,10911 (46.2%)for poverty income ratio, 349 (<0.1%) for smoking, 8885 (37.1%) for drinking,11385 (48.2%) for FBS, 11049 (46.7%) for LDL-C. Numbers not totalling 100% are due to missing data. Dummy variables were used to indicate missing covariate values.

Table S8. Baseline characteristics of patients with CKM syndrome stage 0–4 concerning depression with follow-up data more than 36 months (N = 27,297).

	Depression		
	No	Yes	p-value
N	25053	2244	
Age, years	48.2 ± 18.0	47.9 ± 16.3	0.473
Gender, n (%)			<0.001
Male	12420 (49.6%)	799 (35.6%)	
Female	12633 (50.4%)	1445 (64.4%)	
Race, n (%)			<0.001
Mexican American	3972 (15.9%)	356 (15.9%)	
Other Hispanic	2651 (10.6%)	342 (15.2%)	
Non-Hispanic White	10183 (40.6%)	924 (41.2%)	
Non-Hispanic Black	5356 (21.4%)	475 (21.2%)	
Other Race - Including Multi-Racial	970 (3.9%)	98 (4.4%)	
Non-Hispanic Asian	1921 (7.7%)	49 (2.2%)	
Education Level, n (%)			<0.001
Less Than High School Grad	5945 (23.7%)	828 (36.9%)	
High School Grad/GED or Equivalent	5496 (21.9%)	514 (22.9%)	
Some College or above	12963 (51.7%)	852 (38.0%)	
Marital Status, n (%)			<0.001
Married or Living with partner	14922 (59.6%)	986 (43.9%)	
Separated or Never married	9494 (37.9%)	1209 (53.9%)	
Poverty income ratio, n (%)			<0.001
≤1.30	8204 (32.7%)	1195 (53.3%)	
1.3~1.85	3400 (13.6%)	312 (13.9%)	
> 1.85	11845 (47.3%)	594 (26.5%)	
Smoking, n (%)			<0.001
Never	14240 (56.8%)	908 (40.5%)	
Current smoker	4677 (18.7%)	834 (37.2%)	
Ever smoker	5720 (22.8%)	471 (21.0%)	
Drinking, g/year	23.0 ± 303.5	35.9 ± 305.6	0.053
Albumin, g/L	42.7 ± 3.3	41.9 ± 3.5	<0.001

FBS, mg/dL	108.7 ± 35.8	114.4 ± 44.5	<0.001
HBA1c, %	5.7 ± 1.1	5.9 ± 1.3	<0.001
TC, mg/dL	52.8 ± 16.0	50.8 ± 16.0	<0.001
LDL-C, mg/dL	113.4 ± 35.2	115.7 ± 37.1	0.055
HDL-C, mg/dL	52.8 ± 16.0	50.8 ± 16.0	<0.001
TG, mg/dL	154.2 ± 133.1	172.1 ± 152.2	<0.001
BUN, mg/dL	13.4 ± 5.6	12.7 ± 6.2	<0.001
UACR, mg/g	34.6 ± 656.2	52.6 ± 363.5	0.168
eGFR, ml/min/1.73m2	96.0 ± 22.4	95.6 ± 23.2	0.437
Standing height, cm	167.2 ± 10.2	165.2 ± 9.9	<0.001
Waist circumference, cm	98.8 ± 15.9	102.9 ± 18.0	<0.001
BMI,kg/m²	29.0 ± 6.7	31.1 ± 8.3	<0.001
PHQ9 Score	1.9 ± 2.4	14.3 ± 3.9	<0.001
Physical activity, n (%)	13519 (54.0%)	877 (39.1%)	<0.001
Diabetes, n (%)	2914 (11.6%)	426 (19.0%)	<0.001
Hypertension, n (%)	8418 (33.6%)	1043 (46.5%)	<0.001
Cardiovascular disease, n (%)	1730 (6.9%)	311 (13.9%)	<0.001
Stroke, n (%)	753 (3.0%)	155 (6.9%)	<0.001
Cancer, n (%)	2136 (8.5%)	231 (10.3%)	0.004
Hyperlipidemia,n (%)	7837 (31.3%)	896 (39.9%)	<0.001
Antihypertensive agents,n (%)	7308 (29.2%)	884 (39.4%)	<0.001
Antihyperlipidemic agents,n (%)	5627 (22.5%)	656 (29.2%)	<0.001
Antihyperglycemic agents,n (%)	2119 (72.7%)	302 (70.9%)	0.431
CKD Risk, n (%)			<0.001
Low-risk	22040 (88.0%)	1893 (84.4%)	
Moderate to high-risk	2697 (10.8%)	305 (13.6%)	
Very high-risk	316 (1.3%)	46 (2.0%)	
MetS, n (%)	9000 (35.9%)	1055 (47.0%)	<0.001
Cardiovascular mortality	1730 (6.9%)	311 (13.9%)	<0.001
All-cause mortality	9494 (37.9%)	1209 (53.9%)	<0.001

Frequencies are expressed as absolute numbers and percentages (%); values are means (standard deviation).

Abbreviations: HR, hazard ratio; CI, confidence interval; BMI, body mass index; SBP, systolic blood pressure; DBP, diastolic blood pressure; DM, diabetes mellitus; eGFR, estimated glomerular filtration rate; FBG, fasting blood glucose; HbA1c, hemoglobin A1c; HDL-C, high-density lipoprotein cholesterol; LDL-C, low-density lipoprotein cholesterol; TC, total cholesterol; TG, triglyceride; UACR, urinary albumin creatinine ratio；BUN, blood urea nitrogen; CKD, Chronic kidney disease；MeTS, metabolic syndrome; CKM, Cardiovascular-Kidney-Metabolic Syndrome.

Table S9. Baseline characteristics of patients with CKM syndrome stage 0–4 concerning depression with follow-up data more than 60 months (N = 21,462).

	Depression		
	No	Yes	p-value
N	19655	1807	
Age, years	47.6 ± 17.7	47.2 ± 15.8	0.415
Gender, n (%)			<0.001
Male	12420 (49.6%)	799 (35.6%)	
Female	12633 (50.4%)	1445 (64.4%)	
Race, n (%)			<0.001
Mexican American	3972 (15.9%)	356 (15.9%)	
Other Hispanic	2651 (10.6%)	342 (15.2%)	
Non-Hispanic White	10183 (40.6%)	924 (41.2%)	
Non-Hispanic Black	5356 (21.4%)	475 (21.2%)	
Other Race - Including Multi-Racial	970 (3.9%)	98 (4.4%)	
Non-Hispanic Asian	1921 (7.7%)	49 (2.2%)	
Education Level, n (%)			<0.001
Less Than High School Grad	5945 (23.7%)	828 (36.9%)	
High School Grad/GED or Equivalent	5496 (21.9%)	514 (22.9%)	
Some College or above	12963 (51.7%)	852 (38.0%)	
Marital Status, n (%)			<0.001
Married or Living with partner	14922 (59.6%)	986 (43.9%)	
Separated or Never married	9494 (37.9%)	1209 (53.9%)	
Poverty income ratio, n (%)			<0.001
≤1.30	8204 (32.7%)	1195 (53.3%)	
1.3~1.85	3400 (13.6%)	312 (13.9%)	
> 1.85	11845 (47.3%)	594 (26.5%)	
Smoking, n (%)			<0.001
Never	14240 (56.8%)	908 (40.5%)	
Current smoker	4677 (18.7%)	834 (37.2%)	
Ever smoker	5720 (22.8%)	471 (21.0%)	
Drinking, g/year	25.5 ± 332.2	39.5 ± 338.1	0.086
Albumin, g/L	42.6 ± 3.3	41.7 ± 3.5	<0.001

FBS, mg/dL	107.3 ± 33.9	114.0 ± 45.8	<0.001
HBA1c, %	5.7 ± 1.1	5.9 ± 1.3	<0.001
TC, mg/dL	52.8 ± 16.0	50.8 ± 16.0	<0.001
LDL-C, mg/dL	114.0 ± 34.9	116.5 ± 36.7	0.055
HDL-C, mg/dL	52.4 ± 15.6	50.5 ± 15.5	<0.001
TG, mg/dL	153.4 ± 133.5	173.8 ± 159.2	<0.001
BUN, mg/dL	13.0 ± 5.3	12.3 ± 6.2	<0.001
UACR, mg/g	29.1 ± 703.2	47.3 ± 354.5	0.279
eGFR, ml/min/1.73m2	96.4 ± 21.9	96.4 ± 22.7	0.991
Standing height, cm	167.5 ± 10.2	165.1 ± 9.9	<0.001
Waist circumference, cm	98.5 ± 15.8	102.5 ± 17.9	<0.001
BMI,kg/m²	28.9 ± 6.6	31.0 ± 8.2	<0.001
PHQ9 Score	1.9 ± 2.4	14.3 ± 3.9	<0.001
Physical activity, n (%)	10652 (54.2%)	695 (38.5%)	<0.001
Diabetes, n (%)	2105 (10.7%)	330 (18.3%)	<0.001
Hypertension, n (%)	6393 (32.5%)	830 (45.9%)	<0.001
Cardiovascular disease, n (%)	1239 (6.3%)	237 (13.1%)	<0.001
Stroke, n (%)	539 (2.7%)	115 (6.4%)	<0.001
Cancer, n (%)	1589 (8.1%)	180 (10.0%)	0.004
Hyperlipidemia,n (%)	6023 (30.6%)	697 (38.6%)	<0.001
Antihypertensive agents,n (%)	5485 (27.9%)	702 (38.8%)	<0.001
Antihyperlipidemic agents,n (%)	4186 (21.3%)	503 (27.8%)	<0.001
Antihyperglycemic agents,n (%)	1470 (69.8%)	231 (70.0%)	0.951
CKD Risk, n (%)			<0.001
Low-risk	17524 (89.2%)	1556 (86.1%)	
Moderate to high-risk	1945 (9.9%)	216 (12.0%)	
Very high-risk	186 (0.9%)	35 (1.9%)	
MetS, n (%)	6856 (34.9%)	848 (46.9%)	<0.001
Cardiovascular mortality	379 (1.9%)	37 (2.0%)	0.725
All-cause mortality	1298 (6.6%)	137 (7.6%)	0.111

Frequencies are expressed as absolute numbers and percentages (%); values are means (standard deviation).

Abbreviations: HR, hazard ratio; CI, confidence interval; BMI, body mass index; SBP, systolic blood pressure; DBP, diastolic blood pressure; DM, diabetes mellitus; eGFR, estimated glomerular filtration rate; FBG, fasting blood glucose; HbA1c, hemoglobin A1c; HDL-C, high-density lipoprotein cholesterol; LDL-C, low-density lipoprotein cholesterol; TC, total cholesterol; TG, triglyceride; UACR, urinary albumin creatinine ratio；BUN, blood urea nitrogen; CKD, Chronic kidney disease；MeTS, metabolic syndrome; CKM, Cardiovascular-Kidney-Metabolic Syndrome.

Table S10. Cox proportional hazards regression analysis of PHQ9 score indices concerning all-cause and cardiovascular mortality in a CKM syndrome stage 0–4 population with follow-up data more than 36 months (N = 27,297).

Exposure	Non-adjusted	Adjust I	Adjust II
All-cause mortality			
PHQ-9 score	1.01 (1.00, 1.02) 0.0502	1.05 (1.04, 1.06) <0.0001	1.02 (1.00, 1.03) 0.0062
PHQ-9 score categorical			
<10	Ref.	Ref.	Ref.
>=10, <15	1.12 (0.93, 1.35) 0.2283	1.75 (1.45, 2.10) <0.0001	1.19 (0.97, 1.47) 0.0881
>=15	1.13 (0.90, 1.42) 0.2805	1.77 (1.41, 2.23) <0.0001	1.04 (0.79, 1.36) 0.7970
Exposure	Non-adjusted	Adjust I	Adjust II
Cardiovascular mortality			
PHQ-9 score	1.01 (0.99, 1.03) 0.2989	1.05 (1.03, 1.07) <0.0001	1.02 (1.00, 1.04) 0.0693
PHQ-9 score categorical			
<10	Ref.	Ref.	Ref.
>=10, <15	1.21 (0.88, 1.66) 0.2525	2.05 (1.48, 2.83) <0.0001	1.36 (0.94, 1.95) 0.0982
>=15	0.91 (0.58, 1.44) 0.6919	1.58 (0.99, 2.51) 0.0527	0.97 (0.57, 1.64) 0.8982

Data were presented as HR (95%Cl), p value; Non-adjusted model adjust for: None. Adjust I model adjust for: age; gender; race. Adjust II model adjust for: age; gender; race; education level; marital status; poverty income ratio; smoking; drinking; physical activity; BMI; BUN; HBA1c; FBS; eGFR; hypertension; cardiovascular disease; antihypertensive agents; antihyperglycemic agents; CKD Risk; MeTS; CKM syndrome. Abbreviations: CKM, Cardiovascular Kidney Metabolic Syndrome; Ref., reference.

Table S11. Cox proportional hazards regression analysis of PHQ9 score indices concerning all-cause and cardiovascular mortality in a CKM syndrome stage 0–4 population with follow-up data more than 60 months (N = 21,462).

Exposure	Non-adjusted	Adjust I	Adjust II
All-cause mortality			
PHQ-9 score	1.01 (1.00, 1.02) 0.1643	1.05 (1.04, 1.06) <0.0001	1.02 (1.01, 1.03) 0.0066
PHQ-9 score categorical			
<10	Ref.	Ref.	Ref.
>=10, <15	1.14 (0.91, 1.42) 0.2437	1.86 (1.49, 2.32) <0.0001	1.22 (0.95, 1.56) 0.1125
>=15	1.11 (0.84, 1.47) 0.4470	1.80 (1.36, 2.39) <0.0001	1.11 (0.81, 1.52) 0.5240
Exposure	Non-adjusted	Adjust I	Adjust II
Cardiovascular mortality			
PHQ-9 score	1.00 (0.98, 1.03) 0.6840	1.05 (1.03, 1.07) <0.0001	1.02 (0.99, 1.04) 0.1961
PHQ-9 score categorical			
<10	Ref.	Ref.	Ref.
>=10, <15	1.19 (0.80, 1.78) 0.3827	2.09 (1.40, 3.12) 0.0003	1.42 (0.91, 2.21) 0.1215
>=15	0.81 (0.44, 1.47) 0.4854	1.42 (0.78, 2.60) 0.2551	0.84 (0.42, 1.66) 0.6174

Data were presented as HR (95%Cl), p value; Non-adjusted model adjust for: None. Adjust I model adjust for: age; gender; race. Adjust II model adjust for: age; gender; race; education level; marital status; poverty income ratio; smoking; drinking; physical activity; BMI; BUN; HBA1c; FBS; eGFR; hypertension; cardiovascular disease; antihypertensive agents; antihyperglycemic agents; CKD Risk; MeTS; CKM syndrome. Abbreviations: CKM, Cardiovascular Kidney Metabolic Syndrome; Ref., reference.

Table S12. Cox proportional hazards regression analysis of PHQ9 score indices concerning all-cause and cardiovascular mortality in a CKM syndrome stage 0–4 population (Using Multiple Imputation).

Exposure	Non-adjusted	Adjust I	Adjust II
All-cause mortality			
PHQ-9 score	1.01 (1.00, 1.02) 0.0070	1.04 (1.04, 1.05) <0.0001	1.01 (1.00, 1.02) 0.0173
PHQ-9 score categorical			
<10	Ref.	Ref.	Ref.
>=10, <15	1.13 (0.94, 1.35) 0.1954	1.75 (1.45, 2.10) <0.0001	1.31 (1.08, 1.60) 0.0071
>=15	1.11 (0.88, 1.41) 0.3890	1.68 (1.32, 2.13) <0.0001	0.93 (0.71, 1.22) 0.6096

Exposure	Non-adjusted	Adjust I	Adjust II
Cardiovascular mortality			
PHQ-9 score	1.02 (1.00, 1.03) 0.0499	1.06 (1.04, 1.07) <0.0001	1.02 (1.00, 1.04) 0.0592
PHQ-9 score categorical			
<10	Ref.	Ref.	Ref.
>=10, <15	1.27 (0.93, 1.74) 0.1399	2.15 (1.56, 2.96) <0.0001	1.58 (1.12, 2.22) 0.0088
>=15	0.88 (0.54, 1.43) 0.6063	1.46 (0.90, 2.36) 0.1281	0.77 (0.45, 1.33) 0.3516

Data were presented as HR (95%Cl), p value; Non-adjusted model adjust for: None. Adjust I model adjust for: age; gender; race. Adjust II model adjust for: age; gender; race; education level; marital status; poverty income ratio; smoking; drinking; physical activity; BMI; BUN; HBA1c; FBS; eGFR; hypertension; cardiovascular disease; antihypertensive agents; antihyperglycemic agents; CKD Risk; MeTS; CKM syndrome. Abbreviations: CKM, Cardiovascular Kidney Metabolic Syndrome; Ref., reference.

Table S13 Analysis of the threshold effect of PHQ9 score on all-cause and cardiovascular mortality in patients with CKM syndrome stage 0–4 (Using Multiple Imputation).

PHQ9 score<br>Outcome:	All-cause mortality	Cardiovascular mortality
Model I		
One line effect	1.01 (1.01, 1.02) 0.0029	1.02 (1.00, 1.04) 0.0275
Model II		
Turning point (K)	11	11
< K	1.02 (1.01, 1.04) 0.0006	1.05 (1.02, 1.07) 0.0004
≥ K	0.99 (0.95, 1.02) 0.3895	0.94 (0.87, 1.00) 0.0605
P value for LRT test	0.061	0.006
95% CI for turning point	7, 11	7, 12

Data were presented as HR (95%Cl), *P* value; Model I, linear analysis; Model ll, non-linear analysis. Adjust for: age; gender; race; education level; marital status; poverty income ratio; smoking; drinking; physical activity; BMI; BUN; HBA1c; FBS; eGFR; hypertension; cardiovascular disease; antihypertensive agents; antihyperglycemic agents; CKD Risk; MeTS; CKM syndrome. Abbreviations: HR, hazard ratio; Cl, confidence interval; LRT, logarithm likelihood ratio test.

Table S14 Exploratory Analysis of Potential Pathway Variables in the PHQ-9 and Mortality Association.

**Pathway Variable**	**Total Association (95% CI)**	**P-value**	**Explained Association (95% CI)**	**P-value**	**Independent Association (95% CI)**	**P-value**	**Proportion Explained (95% CI)**
Physical Activity	-31.14 (-48.64, -15.42)	<0.0001	-4.59 (-7.02, -2.67)	<0.0001	-26.55 (-43.13, -11.46)	<0.0001	0.148 (0.080, 0.294)
Poverty Income Ratio	-41.23 (-56.61, -25.51)	<0.0001	-5.74 (-8.69, -3.30)	<0.0001	-35.49 (-51.24, -20.01)	<0.0001	0.139 (0.077, 0.236)
Marital Status	-30.10 (-46.73, -14.01)	<0.0001	-3.87 (-5.83, -2.15)	<0.0001	-26.23 (-42.82, -10.15)	<0.0001	0.128 (0.068, 0.282)

Data were presented as HR (95%Cl), P value. Abbreviations: HR, hazard ratio; Cl, confidence interval.

Model adjust for: age; gender; race; education level; marital status; poverty income ratio; smoking; drinking; physical activity; BUN; HBA1c; FBS; eGFR; hypertension; cardiovascular disease; CKD Risk . Abbreviations: CKM, Cardiovascular Kidney Metabolic Syndrome.

Fig. S1 Association between PHQ9 score and all-cause (A) and cardiovascular mortality (B) in a group with CKM syndrome stage 0–4 with follow-up data more than 36 months.

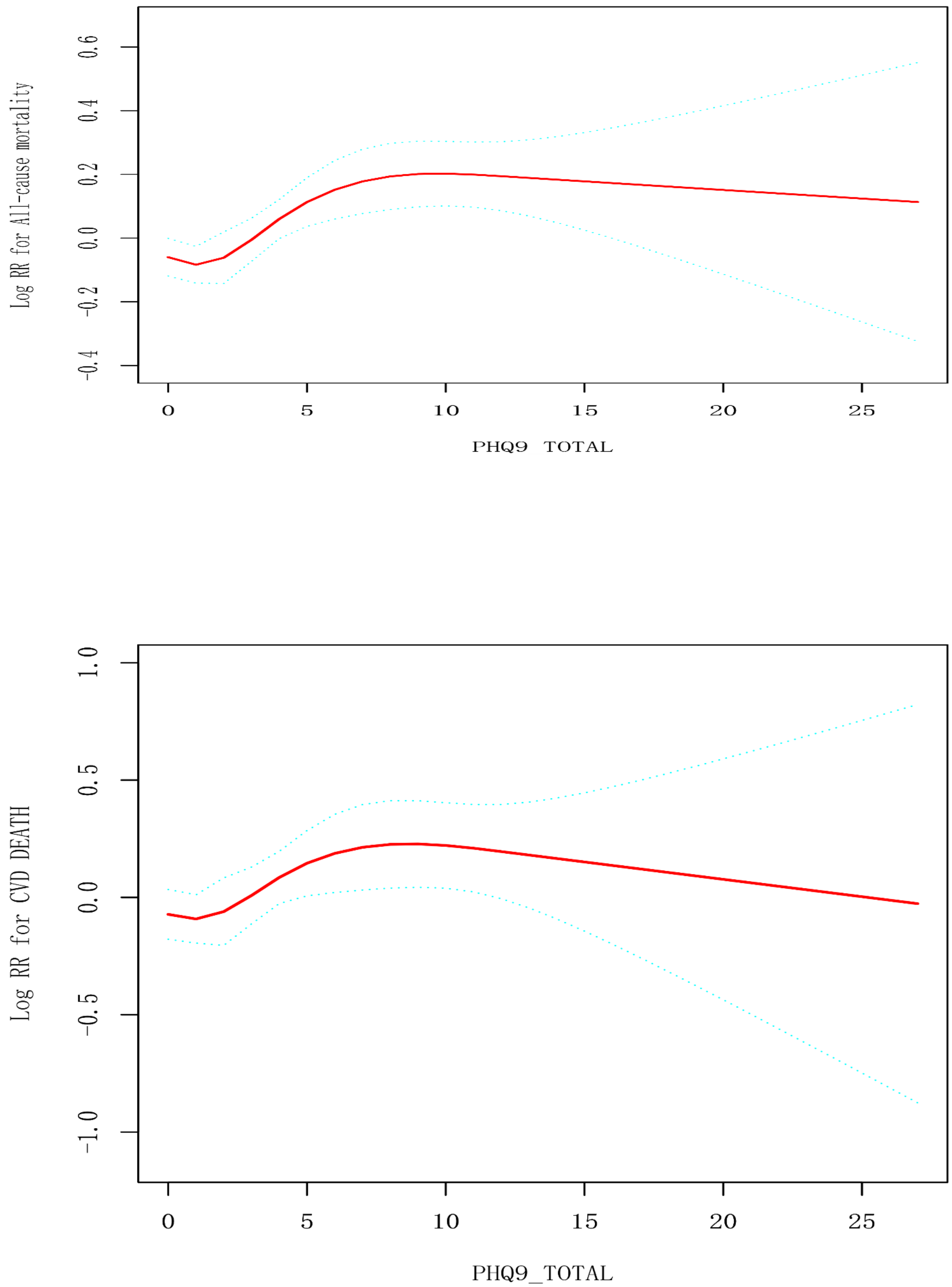

Fig. S2 Association between PHQ9 score and all-cause (A) and cardiovascular mortality (B) in a group with CKM syndrome stage 0–4 with follow-up data more than 60 months.

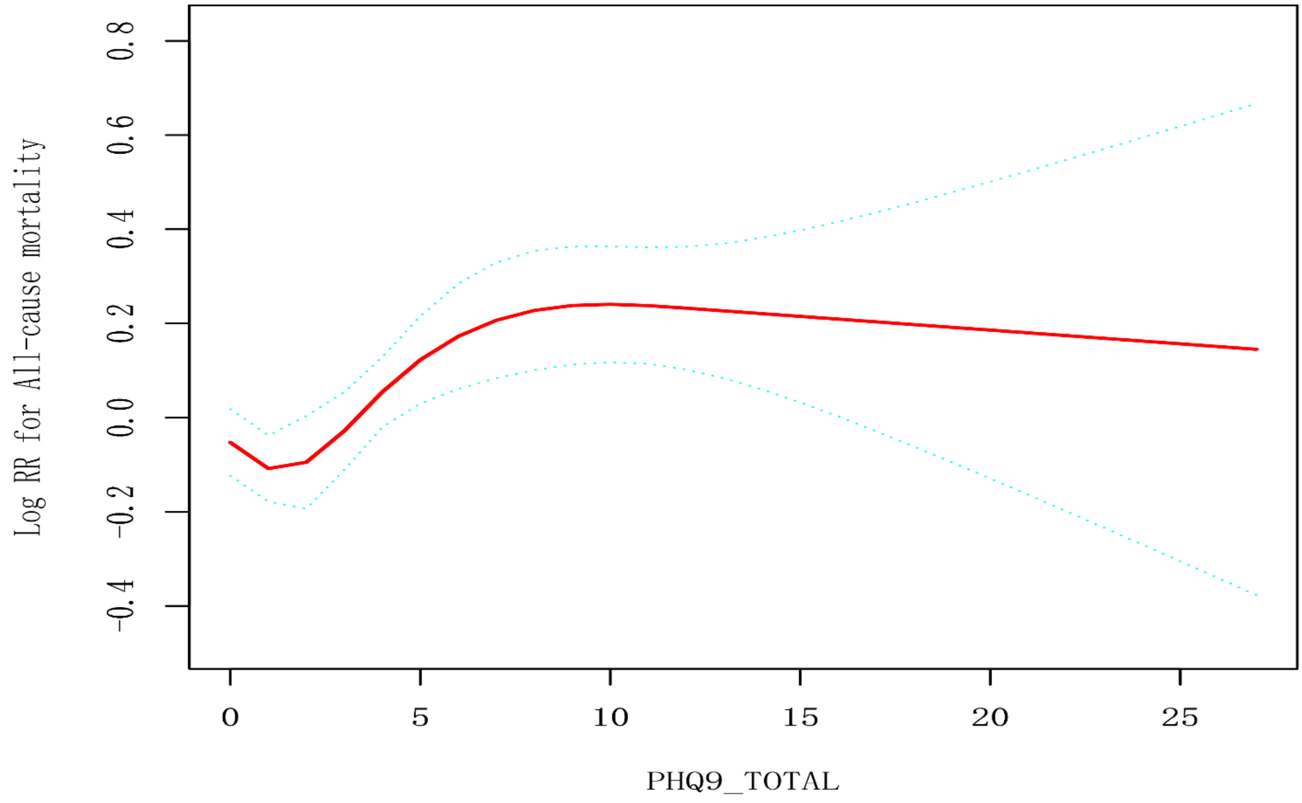

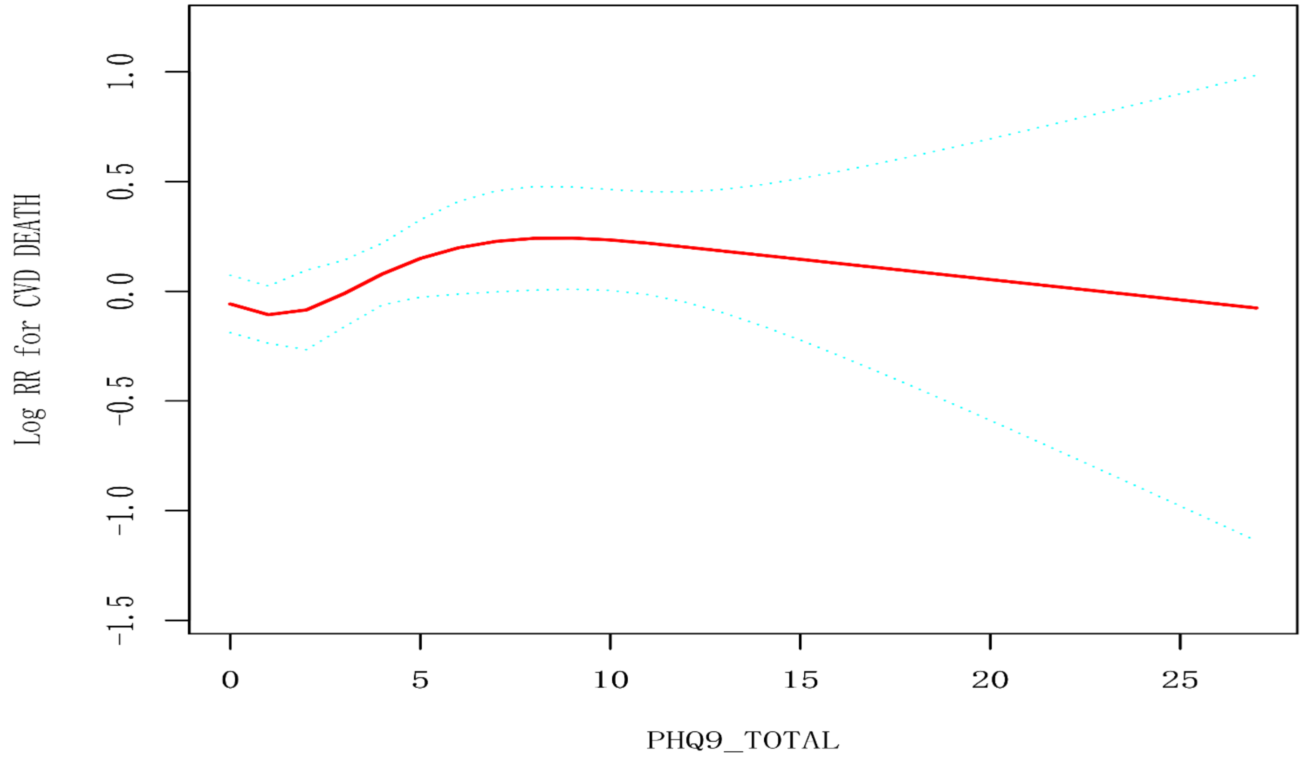

Fig. S3 Association between PHQ9 score and all-cause (A) and cardiovascular mortality (B) in a group with CKM syndrome stage 0–4 (Using Multiple Imputation).

A

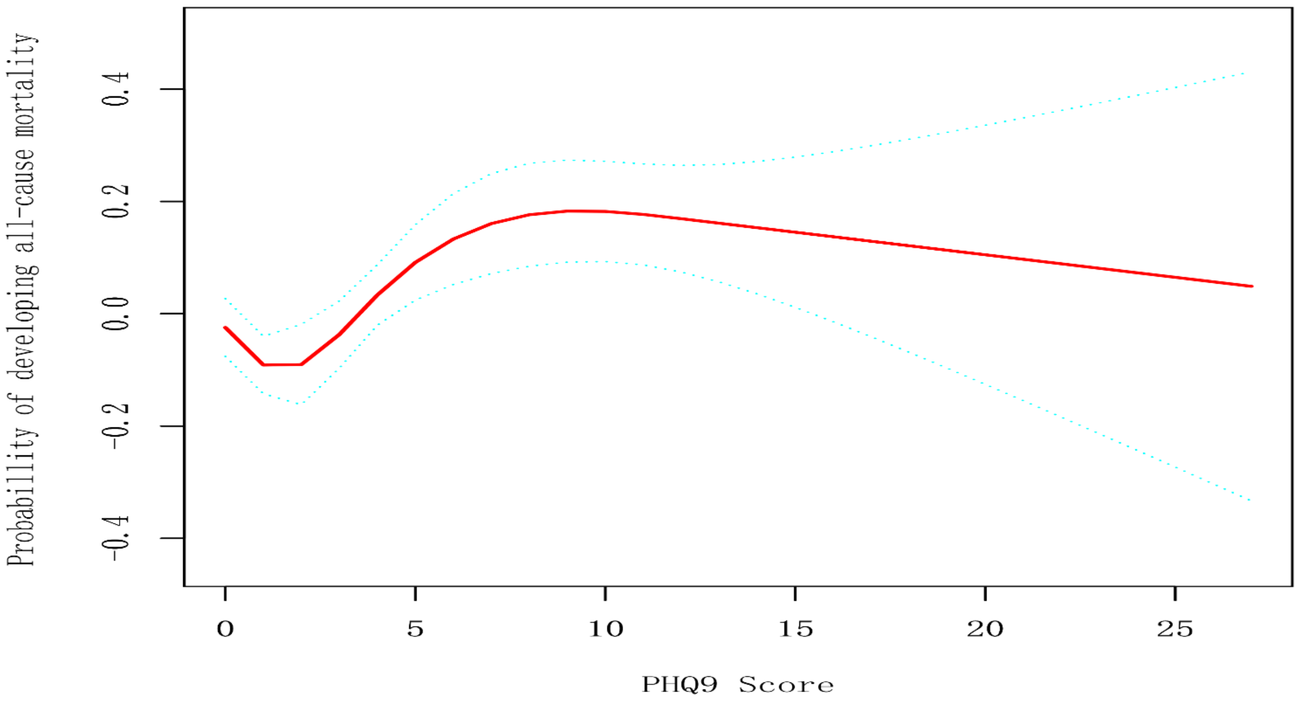

B

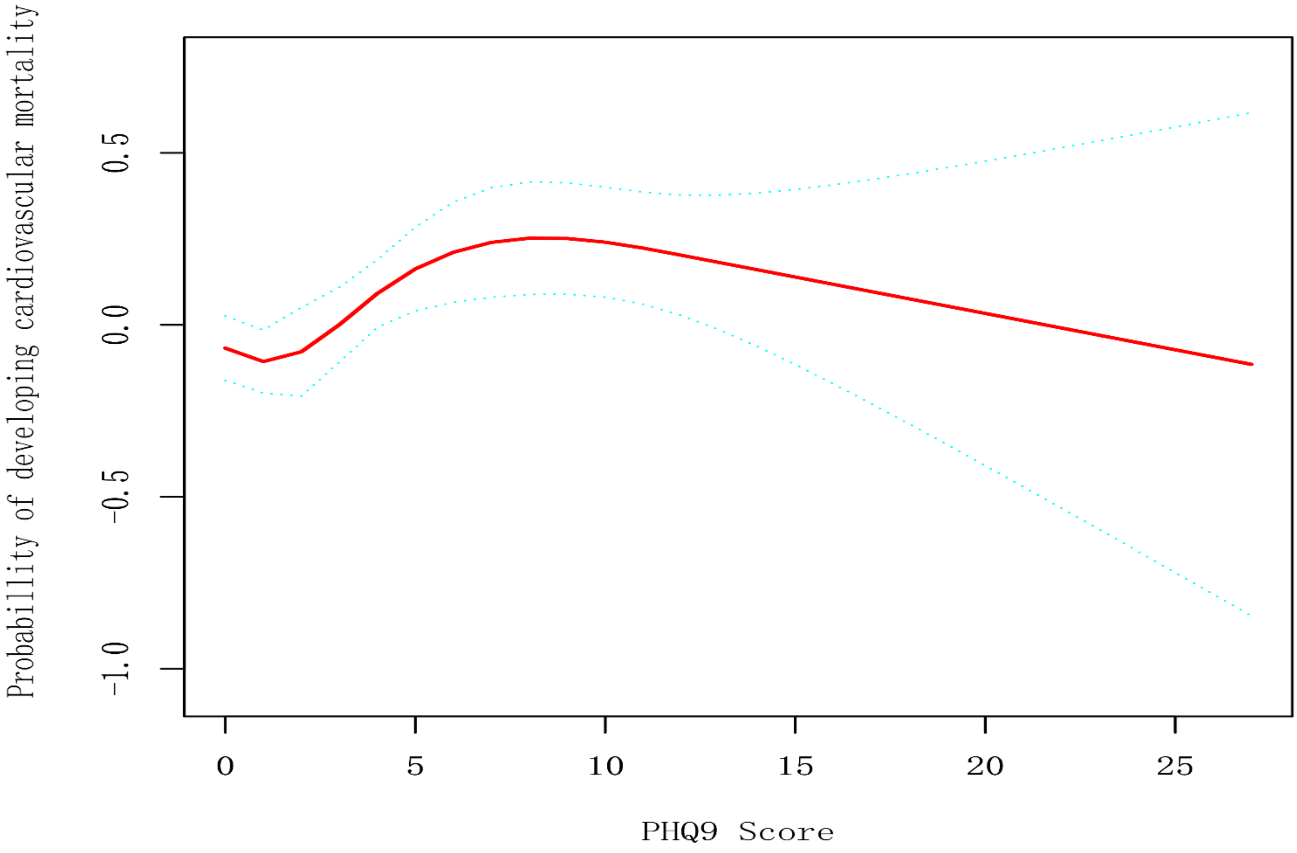

Supplementary Appendix 2

Table S15 Missingness flow table

```
Marital Status: 686 (2.9%) missing values
 Education Level: 700 (3.0%) missing values
 Poverty Income Ratio: 1774 (7.5%) missing values
 BMI: 174 (0.7%) missing values
 UACR, mg/g: 550 (2.3%) missing values
 eGFR, ml/min/1.73m2: 1685 (7.1%) missing values
 TG, mg/dL: 1702 (7.2%) missing values
 Smoking, n (%): 448 (1.9%) missing values
 Albumin, g/L: 1683 (7.1%) missing values
 ALP, U/L: 1688 (7.1%) missing values
 Total calcium, mg/dL: 1716 (7.3%) missing values
 BUN, mg/dL: 1687 (7.1%) missing values
 Standing height, cm: 136 (0.6%) missing values
 Waist circumference, cm: 1389 (5.9%) missing values
 Serum 25(OH) vitamin D, nmol/L: 2194 (9.3%) missing values
 UA, mg/dL: 1691 (7.1%) missing values
 HBA1C: 1348 (5.7%) missing values
 FBG, mg/dL: 14969 (63.3%) missing values
 HLDL.C: 1605 (6.8%) missing values
 TC, mg/dL: 1605 (6.8%) missing values
 LDLC: 15457 (65.4%) missing values
 Cholesterol, mg/dL: 1690 (7.1%) missing values
Creatinine, mg/dL: 1685 (7.1%) missing values
```

Abbreviations: BMI,body mass index;SBP, systolic blood pressure; DBP, diastolic blood pressure; CI,confidence interval; HR, hazard ratio; DM, diabetes mellitus; eGFR; estimated glomerular filtration rate;FBG, fasting blood glucose;HbA1c, hemoglobin A1c; HDL-C, high-density lipoprotein cholesterol; LDL-C, low-density lipoprotein cholesterol; TC, total cholesterol; TG, triglyceride; UACR, urinary albumin creatinine ratio ; ALP, alkaline phosphotase; UA, uric acid; BUN, blood urea nitrogen; CKD , Chronic kidney disease ; MeTS, metabolic syndrome; CKM, Cardiovascular-Kidney-Metabolic Syndrome.

Fig. S4 Directed Acyclic Graph

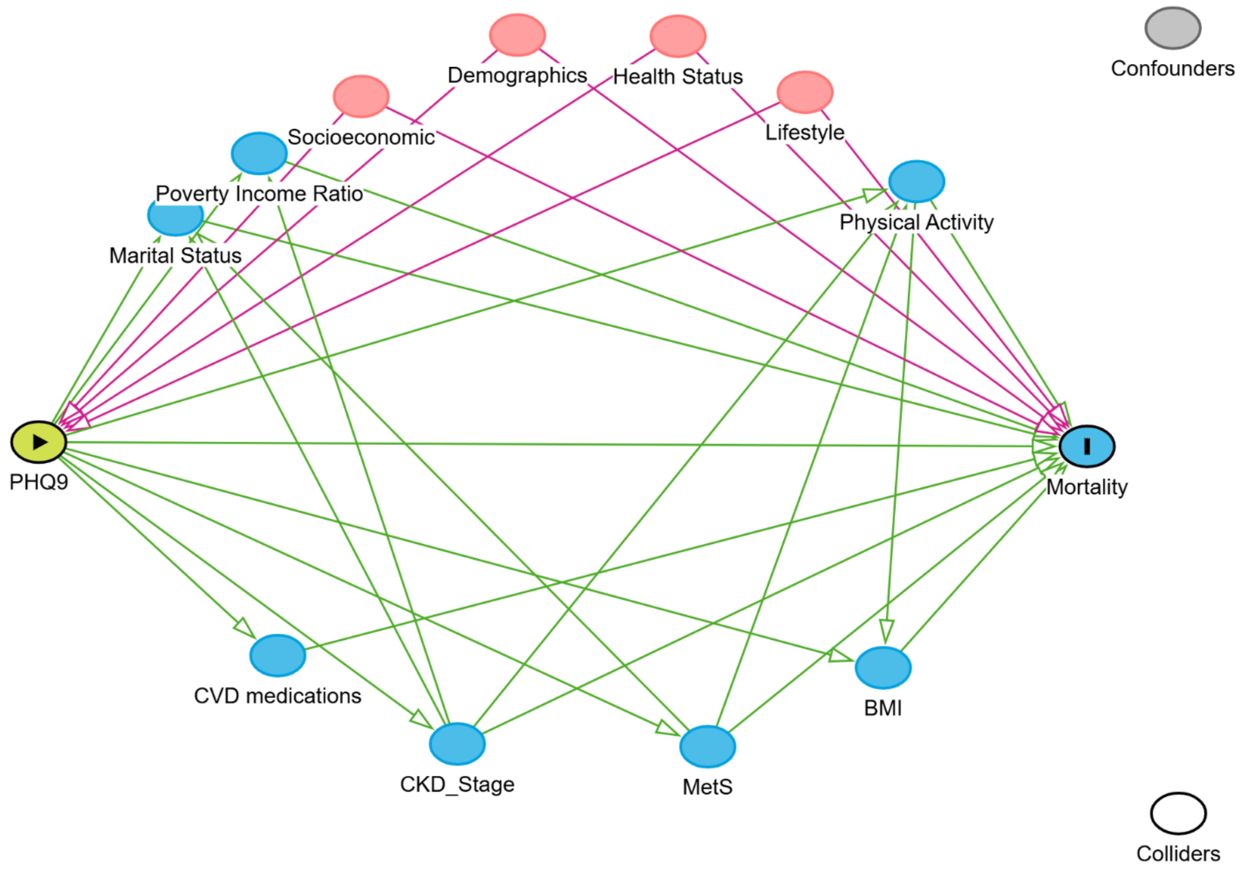

Supplementary Appendix 2

R code for threshold

```
Sys.setlocale(category = 'LC_ALL', locale = 'English_United States.1252');

.libPaths(file.path(R.home(),'library'));

library(doBy);

options(timeout=600);

library(plotrix);

library(stringi);

library(stringr);

library(survival);

library(rms);

library(nnet);

library(car);

library(mgcv);

pdfwd<-6; pdfht<-6;
```

```
load('D:/EmpowerRCH/Analysis/DEP0012/DEP0012_1_tbl/DEP0012_1_tbl_mi.Rdata');
if (length(which(ls()=='EmpowerStatsR'))==0) EmpowerStatsR<-get(ls()[1]);
names(EmpowerStatsR)<-toupper(names(EmpowerStatsR));
originalVNAME<-names(EmpowerStatsR);
ofname<-'DEP0012_1_tbl_4_tbl';
recodevar <- function (var,oldcode,newcode) {
  tmp.v <- var
  nc.tmp <- length(oldcode)
  str <- 0;
  if (!is.numeric(oldcode)) {
    if (sum(grepl('[|(|)|]|^|*|+|$|.|\\|/', oldcode))==0) {
      if (sum(duplicated(substr(oldcode,1,min(nchar(oldcode)))))==0) {oldcode<-paste("^",oldcode,sep=""); str <- 1;}
    }
  }
  for (i in (1:nc.tmp)) {
    if (!is.na(oldcode[i]) & oldcode[i] != "NA") {
      if (str == 1) {tmp.v[str_detect(var, str_escape('(0,19]'))] <- newcode[i];
           } else {tmp.v[var == oldcode[i]]<-newcode[i]; }
         } else if (!is.na(newcode[i])) {tmp.v[is.na(var)] <- newcode[i] }
  }
  if (is.factor(tmp.v)) {tmp.v1<-as.numeric(as.character(tmp.v))} else {tmp.v1<-as.numeric(tmp.v)}
  rm(tmp.v);  return(tmp.v1)
}
attach(EmpowerStatsR);
sink(paste(ofname,'_datastep.lst',sep=''));
print('Creating new variable: MARITAL.STATUS.RCD.RCD');
MARITAL.STATUS.RCD.RCD<- recodevar(MARITAL.STATUS.RCD,c(1,2,NA),c(1,2,3));
summary(MARITAL.STATUS.RCD.RCD);
EmpowerStatsR<-cbind(EmpowerStatsR,MARITAL.STATUS.RCD.RCD);
print('Creating new variable: EDUCATION.LEVEL.RCD.RCD');
EDUCATION.LEVEL.RCD.RCD<- recodevar(EDUCATION.LEVEL.RCD,c(1,3,5,NA),c(1,3,5,6));
summary(EDUCATION.LEVEL.RCD.RCD);
EmpowerStatsR<-cbind(EmpowerStatsR,EDUCATION.LEVEL.RCD.RCD);
print('Creating new variable: DIABETES_TREATMENT.RCD');
```

```
DIABETES_TREATMENT.RCD<- recodevar(DIABETES_TREATMENT,c(0,1,NA),c(0,1,0));

summary(DIABETES_TREATMENT.RCD);

EmpowerStatsR<-cbind(EmpowerStatsR,DIABETES_TREATMENT.RCD);

print('Creating new variable: CVD.RCD');

CVD.RCD<- recodevar(UCOD_LEADING,c(1,2,3,4,5,6,7,8,9,10,NA),c(1,0,0,0,1,0,0,0,0,0,0));

summary(CVD.RCD);

EmpowerStatsR<-cbind(EmpowerStatsR,CVD.RCD);

print('Creating new variable: INDFMMPC.RCD');

INDFMMPC.RCD<- recodevar(INDFMMPC,c(1,2,3,7,9,NA),c(1,2,3,9,9,9));

summary(INDFMMPC.RCD);

EmpowerStatsR<-cbind(EmpowerStatsR,INDFMMPC.RCD);

print('Creating new variable: SMOKER.RCD');

SMOKER.RCD<- recodevar(SMOKER,c(0,1,2,NA),c(0,1,2,3));

summary(SMOKER.RCD);

EmpowerStatsR<-cbind(EmpowerStatsR,SMOKER.RCD);

rm(MARITAL.STATUS.RCD.RCD,EDUCATION.LEVEL.RCD.RCD,DIABETES_TREATMENT.RCD,CVD.RCD,INDFMMPC.RCD,S
MOKER.RCD);

sink();

vname<-
c(NA,'MI.ITER','MI.ITER.0','MI.ITER.1','MI.ITER.2','MI.ITER.3','MI.ITER.4','MI.ITER.5','AGE','GENDER','GENDER.1','GEND
ER.2','RACE','RACE.1','RACE.2','RACE.3','RACE.4','RACE.5','RACE.6','FBG','BMXBMI','LBXSBU','HBA1C','MORTSTAT','MO
RTSTAT.0','MORTSTAT.1','UCOD_LEADING','UCOD_LEADING.1','UCOD_LEADING.2','UCOD_LEADING.3','UCOD_LEADI
NG.4','UCOD_LEADING.5','UCOD_LEADING.6','UCOD_LEADING.7','UCOD_LEADING.8','UCOD_LEADING.9','UCOD_LEA
DING.10','PERMTH_INT','INDFMMPC','INDFMMPC.1','INDFMMPC.2','INDFMMPC.3','INDFMMPC.7','INDFMMPC.9','H
EART_DISEASE_HISTORY','HEART_DISEASE_HISTORY.0','HEART_DISEASE_HISTORY.1','HYPERTENSION_HISTORY','HYP
ERTENSION_HISTORY.0','HYPERTENSION_HISTORY.1','HYPERLIPIDEMIA_HISTORY','HYPERLIPIDEMIA_HISTORY.0','HYP
ERLIPIDEMIA_HISTORY.1','DIABETES_TREATMENT','DIABETES_TREATMENT.0','DIABETES_TREATMENT.1','SMOKER','S
MOKER.0','SMOKER.1','SMOKER.2','EGFR2021','METS','METS.0','METS.1','CKD_RISK','CKD_RISK.0','CKD_RISK.1','CKD_
RISK.2','CKM_STAGE','CKM_STAGE.0','CKM_STAGE.1','CKM_STAGE.2','CKM_STAGE.3','CKM_STAGE.4','PHQ9_TOTAL',
'ACTIVITY.STAT.ANY','ACTIVITY.STAT.ANY.0','ACTIVITY.STAT.ANY.1','MARITAL.STATUS.RCD','MARITAL.STATUS.RCD.1',
'MARITAL.STATUS.RCD.2','EDUCATION.LEVEL.RCD','EDUCATION.LEVEL.RCD.1','EDUCATION.LEVEL.RCD.3','EDUCATIO
N.LEVEL.RCD.5','MARITAL.STATUS.RCD.RCD','MARITAL.STATUS.RCD.RCD.1','MARITAL.STATUS.RCD.RCD.2','MARITAL.
STATUS.RCD.RCD.3','EDUCATION.LEVEL.RCD.RCD','EDUCATION.LEVEL.RCD.RCD.1','EDUCATION.LEVEL.RCD.RCD.3','ED
UCATION.LEVEL.RCD.RCD.5','EDUCATION.LEVEL.RCD.RCD.6','DIABETES_TREATMENT.RCD','DIABETES_TREATMENT.RC
D.0','DIABETES_TREATMENT.RCD.1','CVD.RCD','CVD.RCD.0','CVD.RCD.1','INDFMMPC.RCD','INDFMMPC.RCD.1','INDF
MMPC.RCD.2','INDFMMPC.RCD.3','INDFMMPC.RCD.9','SMOKER.RCD','SMOKER.RCD.0','SMOKER.RCD.1','SMOKER.RC
D.2','SMOKER.RCD.3')[-1];

vlabel<-c(NA,'MI.ITER',' 0',' 1',' 2',' 3',' 4',' 5','AGE','GENDER',' 1',' 2','RACE',' 1',' 2',' 3',' 4',' 5','
6','FBG','BMXBMI','LBXSBU','HBA1C','MORTSTAT',' 0',' 1','UCOD_LEADING',' 1',' 2',' 3',' 4',' 5',' 6',' 7',' 8',' 9','
10','PERMTH_INT','INDFMMPC',' 1',' 2',' 3',' 7',' 9','HEART_DISEASE_HISTORY',' 0',' 1','HYPERTENSION_HISTORY','
0',' 1','HYPERLIPIDEMIA_HISTORY',' 0',' 1','DIABETES_TREATMENT',' 0',' 1','SMOKER',' 0',' 1','
2','EGFR2021','METS',' 0',' 1','CKD_RISK',' 0',' 1',' 2','CKM_STAGE',' 0',' 1',' 2',' 3','
4','PHQ9_TOTAL','ACTIVITY.STAT.ANY',' 0',' 1','MARITAL.STATUS.RCD',' 1',' 2','EDUCATION.LEVEL.RCD',' 1',' 3','
```

```
5','MARITAL.STATUS.RCD recoded',' 1',' 2',' 3','EDUCATION.LEVEL.RCD recoded',' 1',' 3',' 5','
6','DIABETES_TREATMENT recoded',' 0',' 1','CVD DEATH',' 0',' 1','INDFMMPC recoded',' 1',' 2',' 3',' 9','SMOKER
recoded',' 0',' 1',' 2',' 3')[-1];

varused4this <-
c('MI.ITER','AGE','GENDER','RACE','FBG','BMXBMI','LBXSBU','HBA1C','MORTSTAT','UCOD_LEADING','PERMTH_INT','IN
DFMMPC','HEART_DISEASE_HISTORY','HYPERTENSION_HISTORY','HYPERLIPIDEMIA_HISTORY','DIABETES_TREATMEN
T','SMOKER','EGFR2021','METS','CKD_RISK','CKM_STAGE','PHQ9_TOTAL','ACTIVITY.STAT.ANY','MARITAL.STATUS.RCD'
,'EDUCATION.LEVEL.RCD','MARITAL.STATUS.RCD.RCD','EDUCATION.LEVEL.RCD.RCD','DIABETES_TREATMENT.RCD','C
VD.RCD','INDFMMPC.RCD','SMOKER.RCD');

pkgs<-c('MASS','gdata','survival');

for (g in pkgs) {

if (!(g %in% rownames(installed.packages()))) install.packages(g,repos='https://cloud.r-project.org');

}

library(MASS);

library(gdata);

library(survival);

WD <- EmpowerStatsR; rm(EmpowerStatsR); gc();

title<-'阈值效应分析';

WD<-subset(WD, ((!is.na(MI.ITER) & (MI.ITER == 1))));

wd.subset<-paste("Use subset of data: (!is.na(MI.ITER) & (MI.ITER == 1))");

weights.var<- NA;

yvname<-c('MORTSTAT','CVD.RCD');

ydist<-c('breslow','breslow');

ylink<-c('0','0');

ylv<-c(2,2);

par1<-'自动寻找最佳拐点';

avname<-
c('AGE','GENDER','RACE','INDFMMPC.RCD','SMOKER.RCD','DIABETES_TREATMENT.RCD','EDUCATION.LEVEL.RCD.RCD'
,'MARITAL.STATUS.RCD.RCD','ACTIVITY.STAT.ANY','BMXBMI','FBG','LBXSBU','HBA1C','EGFR2021','HEART_DISEASE_HI
STORY','HYPERTENSION_HISTORY','HYPERLIPIDEMIA_HISTORY','METS','CKD_RISK','CKM_STAGE');

saf<-c(0,0,0,0,0,0,0,0,0,0,0,0,0,0,0,0,0,0,0,0);

alv<-c(0,2,6,4,4,2,4,3,2,0,0,0,0,0,2,2,2,2,3,5);

bvar<- NA;

xvname<-c('PHQ9_TOTAL');

xlv<-c(0);

chk<- 1;

cox<- 1;

timevar<-'PERMTH_INT'; timevlv<- 0;
```

```
vname.start<- NA;

subjvname<- NA;

gee.TYPE<-NA;

prn<-1;

dec<-2;

##R package## MASS gdata survival ##R package##;

pvformat<-function(p,dec) {

  pp <- sprintf(paste("%.",dec,"f",sep=""),as.numeric(p))

  if (is.matrix(p)) {pp<-matrix(pp, nrow=nrow(p)); colnames(pp)<-colnames(p);rownames(pp)<-rownames(p);}

  lw <- paste("<",substr("0.00000000000",1,dec+1),"1",sep="");

  pp[as.numeric(p)<(1/10^dec)]<-lw

  return(pp)

}

numfmt<-function(p,dec) {

  if (is.list(p)) p<-as.matrix(p)

  pp <- sprintf(paste("%.",dec,"f",sep=""),as.numeric(p))

  if (is.matrix(p)) {pp<-matrix(pp, nrow=nrow(p));colnames(pp)<-colnames(p);rownames(pp)<-rownames(p);}

  pp[as.numeric(p)>10000000]<- "inf."

  pp[is.na(p) | gsub(" ","",p)==""]<- ""

  pp[p=="-Inf"]<-"-Inf"

  pp[p=="Inf"]<-"Inf"

  return(pp)

}

mat2htmltable<-function(mat) {

  t1<- apply(mat,1,function(z) paste(z,collapse="</td><td>"))

  t2<- paste("<tr><td>",t1,"</td></tr>")

  return(paste(t2,collapse=" "))

}

setcox<-function(fmlx, i, wdtmp) {

  if (clogit==1) {

    fml<-paste(yvname[i],"~",fmlx,"+strata(",subjvname,")",sep="")

    mdl<-try(clogit(formula(fml),weights=wdtmp$weights,data=wdtmp,na.action=na.omit,method="exact"))

  } else {

    fml<-paste(fmlt,yvname[i],")~",fmlx);
```

```
    mdl<-try(coxph(formula(fml),weights=wdtmp$weights,data=wdtmp,na.action=na.omit,method=ydist[i]))
  }
  return(mdl)
}
mdl2oo<-function(mdl, xxname) {
 if (substr(mdl[1],1,5)!="Error") {
  decp<-dec+2; if (decp>4) decp<-4
  gs<-summary(mdl); print(mdl$formula); print(gs)
  gsparm <- gs$coefficients;
  gsp<-gsparm[match(xxname,rownames(gsparm)),]
  if (length(xxname)==1) {beta<-gsp[1]; se<-gsp[3]; pv<-gsp[5];
  } else {beta<-gsp[,1]; se<-gsp[,3]; pv<-gsp[,5]; }
  ci1<- beta-1.96*se; ci2<- beta+1.96*se
  pvx<-substr(rep("****",length(pv)),1,(pv<=0.05)+(pv<=0.01)+(pv<=0.001))
  if (colprn==3) {pvv<-pvx;} else {pvv<-pvformat(pv,decp);}
  o1<-paste(numfmt(exp(beta),dec)," (",numfmt(exp(ci1),dec),", ",numfmt(exp(ci2),dec),")",sep="")
  o1<-paste(o1,pvv); o1[is.na(beta)]<-NA
 } else {o1<-rep(NA,length(xxname));}
 return(o1)
}
removeNA<-function(i,j,wdf) {
 vvv<-c(yvname[i],xvname[j],avname,subjvname,bvar,vname.start,timevar);
 vvv<-vvv[!is.na(vvv)]; vvv<-vvv[vvv>" "]
 tmp<-is.na(wdf[,vvv]);
 return(wdf[apply(tmp,1,sum)==0,])
}
get.tpval<-function(i,j,g,wdtmp,tppmin=NA,tppmax=NA) {
  if (sum(is.na(wdtmp))>0) {
    if (is.na(g)) {wdtmp<-removeNA(i,j,WD);
    } else if (g<nblv) {wdtmp0<-WD[WD[,bvar]==blv[g],]; wdtmp<-removeNA(i,j,wdtmp0);
    } else {wdtmp<-removeNA(i,j,WD); }
  }
  if (is.na(g)) {fmladj1<-fmladj;
  } else if (g<nblv) {fmladj1<-fmladj;
```

```
} else {fmladj1<-paste(fmladj,"+factor(",bvar,")",sep="");}
xTMP <- wdtmp[,xvname[j]]
tmp.ss<-seq(0.05,0.95,0.05)
tp<-quantile(xTMP,probs=tmp.ss,na.rm=TRUE)
tmp.llk<-rep(NA,length(tmp.ss))
fml<-paste(xvname[j],"+tmp.X",fmladj1)
if (!is.na(tppmin) & !is.na(tppmax)) {
  tp2.min = tppmin; tp2.max = tppmax;
} else {
  for (k in (1:length(tmp.ss))) {
    tmp.X<-(xTMP > tp[k])*(xTMP-tp[k]); wdtmp1<-cbind(wdtmp,tmp.X)
    tmp.mdl<-setcox(fml, i, wdtmp1);
    tmp.llk[k]<-tmp.mdl$loglik[2];
    rm(wdtmp1, tmp.X)
  }
  tp1<-tmp.ss[which.max(tmp.llk)]
  tp2.min = tp1 - 0.04
  tp2.max = tp1 + 0.04
  if (tp2.min<0.05) {tp2.min=0.05}
  if (tp2.max>0.95) {tp2.max=0.95}
}
tp.pctlrange<-quantile(xTMP,probs=c(tp2.min,tp2.max),na.rm=TRUE)
tp.range<-unique(xTMP[xTMP>tp.pctlrange[1] & xTMP<tp.pctlrange[2]])
while (length(tp.range)>5) {
  tmp.pct3<-quantile(tp.range,probs=c(0,0.25,0.5,0.75,1),type=3)
  tmp.llk3<-rep(NA,3)
  for (k in (2:4)) {
    tmp.X<-(xTMP>tmp.pct3[k])*(xTMP-tmp.pct3[k]); wdtmp1<-cbind(wdtmp,tmp.X)
    tmp.mdl<-setcox(fml, i, wdtmp1);
    tmp.llk3[k-1]<-tmp.mdl$loglik[2];
    rm(wdtmp1, tmp.X)
   }
   tmp.min3<-which.max(tmp.llk3)
   tp.range<-tp.range[tp.range>=tmp.pct3[tmp.min3] & tp.range<=tmp.pct3[tmp.min3+2]]
```

```
 }
 if (length(tp.range)>0) {
  if (length(tp.range)==1) {tp.val=tp.range[1];} else {
   tmp.llk<-rep(NA,length(tp.range))
   for (k in (1:length(tp.range))) {
    tmp.X<-(xTMP>tp.range[k])*(xTMP-tp.range[k]); wdtmp1<-cbind(wdtmp,tmp.X)
    tmp.mdl<-setcox(fml, i, wdtmp1);
    tmp.llk[k]<-tmp.mdl$loglik[2];
    rm(wdtmp1, tmp.X)
   }
   tp.val<-tp.range[which.max(tmp.llk)]
  }
 } else { tp.val<-tp.pctlrange[1];}
 return(round(tp.val,dec));
}
get2lines<-function(i,j,g,tp.value) {
 if (is.na(g)) {fmladj1<-fmladj;wdtmp<-removeNA(i,j,WD);
 } else if (g<nblv) {fmladj1<-fmladj;wdtmp0<-WD[WD[,bvar]==blv[g],];wdtmp<-removeNA(i,j,wdtmp0);
 } else {fmladj1<-paste(fmladj,"+factor(",bvar,")",sep="");wdtmp<-removeNA(i,j,WD);}
 xTMP<-wdtmp[,xvname[j]]
 tmp.X1<-(xTMP<=tp.value)*(xTMP-tp.value)
 tmp.X2<-(xTMP> tp.value)*(xTMP-tp.value)
 wdtmp1<-cbind(wdtmp,xTMP,tmp.X1,tmp.X2)
 fml0<-paste("xTMP+tmp.X2",fmladj1)
 fml1<-paste("tmp.X1+tmp.X2",fmladj1)
 fml2<-paste("xTMP",fmladj1)
 fmlp<-paste("xTMP+tmp.X2")
 tmpn<-nrow(wdtmp);
 tmp.mdl0<-setcox(fml0,i,wdtmp1); tmp.mdl1<-setcox(fml1,i,wdtmp1); tmp.mdl2<-setcox(fml2,i,wdtmp1)
 m2<-mdl2oo(tmp.mdl2,"xTMP")
 m1<-mdl2oo(tmp.mdl1,c("tmp.X1","tmp.X2"))
 m0<-mdl2oo(tmp.mdl0,"tmp.X2")
 plrt<-pvformat(anova(tmp.mdl0,tmp.mdl2)$"P(>|Chi|)"[2],3)
 plrti<-""; plrtx<-""
```

```
  if (!is.na(g) & g==nblv) {
    fmli<-paste("xTMP+xTMP*factor(",bvar,")",fmladj)
    fmlx<-paste("tmp.X1+tmp.X2+tmp.X1*factor(",bvar,")+tmp.X2*factor(",bvar,")",fmladj)
    tmp.mdli<-setcox(fmli,i,wdtmp1);
    tmp.mdlx<-setcox(fmlx,i,wdtmp1);
    plrti<-paste("P-interaction:", pvformat(anova(tmp.mdli,tmp.mdl2)$"P(>|Chi|)"[2],3))
    plrtx<-paste("P-interaction:", pvformat(anova(tmp.mdlx,tmp.mdl1)$"P(>|Chi|)"[2],3))
  }
  oo<-list(c(plrti,m2,plrtx,tp.value,m1,m0,plrt),tmpn)
  return(oo)
}
get3lines<-function(i,j,g,tp.value) {
  if (is.na(g)) {fmladj1<-fmladj;wdtmp<-removeNA(i,j,WD);
  } else if (g<nblv) {fmladj1<-fmladj;wdtmp0<-WD[WD[,bvar]==blv[g],];wdtmp<-removeNA(i,j,wdtmp0);
  } else {fmladj1<-paste(fmladj,"+factor(",bvar,")",sep="");wdtmp<-removeNA(i,j,WD);}
  xTMP<-wdtmp[,xvname[j]]; tp1<-tp.value[1]; tp2<-tp.value[2]
  tmp.X1<- (xTMP< tp1)*(xTMP-tp1)
  tmp.X2<-((xTMP>=tp1) & (xTMP<=tp2))*(xTMP-tp1)
  tmp.X3<- (xTMP> tp2)*(xTMP-tp2)
  tmp.B1<- (xTMP>tp2)
  wdtmp1<-cbind(wdtmp,xTMP,tmp.X1,tmp.X2,tmp.X3,tmp.B1)
  fml0<-paste("xTMP+tmp.X1+tmp.X3+tmp.B1",fmladj1)
  fml1<-paste("tmp.X1+tmp.X2+tmp.X3+tmp.B1",fmladj1)
  fml2<-paste("xTMP",fmladj1)
  tmpn<-nrow(wdtmp)
  tmp.mdl0<-setcox(fml0,i,wdtmp1); tmp.mdl1<-setcox(fml1,i,wdtmp1); tmp.mdl2<-setcox(fml2,i,wdtmp1);
  m2<-mdl2oo(tmp.mdl2,"xTMP")
  m1<-mdl2oo(tmp.mdl1,c("tmp.X1","tmp.X2","tmp.X3"))
  m0<-mdl2oo(tmp.mdl0,c("tmp.X1","tmp.X3"))
  plrt<-pvformat(anova(tmp.mdl0,tmp.mdl2)$"P(>|Chi|)"[2],3)
  plrti<-""; plrtx<-""
  if (!is.na(g) & g==nblv) {
    fmli<-paste("xTMP+xTMP*factor(",bvar,")",fmladj)
```

```
    fmlx<-
paste("tmp.X1+tmp.X2+tmp.X3+tmp.X1*factor(",bvar,")+tmp.X2*factor(",bvar,")+tmp.X3*factor(",bvar,")+tmp.B1",f
mladj)
    tmp.mdli<-setcox(fmli,i,wdtmp1);
    tmp.mdlx<-setcox(fmlx,i,wdtmp1);
    plrti<-paste("P-interaction:", pvformat(anova(tmp.mdli,tmp.mdl2)$"P(>|Chi|)"[2],3))
    plrtx<-paste("P-interaction:", pvformat(anova(tmp.mdlx,tmp.mdl1)$"P(>|Chi|)"[2],3))
  }
  oo<-list(c(plrti,m2,plrtx,paste(tp.value,collapse=", "),m1,m0,plrt),tmpn)
  return(oo)
}
getci4tp<-function(i,j,g,tp0=NA) {
  set.seed(123456)
  if (is.na(g)) {wdt<-removeNA(i,j,WD);
  } else if (g<nblv) {wdt<-WD[WD[,bvar]==blv[g],];wdt<-removeNA(i,j,wdt);
  } else {wdt<-removeNA(i,j,WD);}
  nnwd<-nrow(wdt); tp.vv<-rep(NA,1000)
  if (!is.na(tp0)) {
    tpp0 = sum(wdt[,xvname[j]] < tp0)/length(wdt[,xvname[j]])
    tppmin = max(tpp0 - 0.09, 0.05)
            tppmax = min(tpp0 + 0.09, 0.95)
  } else {
    tppmin = NA; tppmax = NA
  }
  for (s in (1:1000)) {
    WDi<-wdt[sample(1:nnwd,nnwd,replace=T),]
    tp.vv[s]<-get.tpval(i,j,NA,WDi, tppmin, tppmax); rm(WDi)
  }
  tpci<-paste(quantile(tp.vv,probs=c(0.025,0.975)),collapse=", ")
  return(tpci);
}
if (!is.na(weights.var)) {weights<-WD[,weights.var];} else {weights<-1;}
WD<-cbind(WD,weights);
vlabelN<-(substr(vlabel,1,1)==" ");
vlabelZ<-vlabel[vlabelN];vlabelV<-vlabel[!vlabelN]
```

```
vnameV<-vname[!vlabelN];vnameZ<-vname[vlabelN]
allvname<-c(yvname,xvname,bvar,avname,subjvname,vname.start,timevar);
allvname<-allvname[!is.na(allvname)]
WD<-WD[,allvname];
w<-c("<!DOCTYPE html><html lang='zh'><head><meta charset='utf-8'></head><body>")
w<-c(w,paste("<h2>", title, "</h2>"))
if (length(avname)>0)  {
  if (sum((saf=="s" | saf=="S") & alv>0)>0) w<-c(w,"</br>Spline smoothing only applies for continuous variables")
  if (!is.na(subjvname)) saf<-rep(0,length(saf))
}
if (sum(xlv>0)>0) w<-c(w,"Categorical exposure variables were ignored")
xvname<-xvname[xlv==0];
if (!is.na(subjvname)) WD<-WD[order(WD[,subjvname]),];
fmladj<-""; avb=""; smoothav<-0;
if (length(avname)>0) {
  avb<-vlabelV[match(avname,vnameV)];
  avname_ <- avname
  smoothavi<-((saf=="s" | saf=="S") & alv==0)
  smoothav<-sum(smoothavi)
  avname_[smoothavi]<-paste("rcs(",avname[smoothavi],")",sep="")
  avb1<-avb
  avb1[smoothavi]<-paste(avb[smoothavi],"(Smooth)",sep="")
  avname_[alv>0]<-paste("factor(",avname[alv>0],")",sep="")
  fmladj<-paste("+",paste(avname_,collapse="+"))
}
if (is.na(bvar)) {
  blvb<-"N"; blvb_<-"N"; nblv<-1; blbl<-"";
} else {
  blbl<-vlabelV[match(bvar,vnameV)]; if (is.na(blbl)) blbl<-bvar;
  blv<-levels(factor(WD[,bvar])); nblv<-length(blv)+1
  blvb_<-vlabelZ[match(paste(bvar,".",blv,sep=""),vnameZ)];
  blvb_[is.na(blvb_)]<-blv[is.na(blvb_)];
  blvb<-c(paste(blbl,blvb_,sep="="),"Total");
  blvb_<-c(blvb_,"Total")
```

```
  WD<-WD[!is.na(WD[,bvar]),]
}
ny=length(yvname); nx=length(xvname);
xb<-vlabelV[match(xvname,vnameV)]; xb[is.na(xb)]<-xvname[is.na(xb)]
yb<-vlabelV[match(yvname,vnameV)]; yb[is.na(yb)]<-yvname[is.na(yb)]
if (!is.na(vname.start)) {
  fmlt<-paste("Surv(,",vname.start,",",timevar,",",sep="");
  WD<-WD[!is.na(WD[,vname.start]) & !is.na(WD[,timevar]),]
} else {
  if (!is.na(timevar)) {
    fmlt<-paste("Surv(",timevar,",",sep="");
    WD<-WD[!is.na(WD[,timevar]),]
  }
}
colprn<-prn
clogit<-0;
if (!is.na(subjvname) & is.na(timevar)) clogit<-1
if (is.na(par1)) par1<-"";
if (is.numeric(par1)) {tp.vv<-par1;
} else {
  tmp<-as.numeric(strsplit(par1," ")[[1]]);  tp.vv<-c(tmp[!is.na(tmp)],NA)
}
prn<-ifelse(!is.na(bvar), "S", ifelse(nx>1 & ny==1, "X", "Y"));

if (length(tp.vv)>2) tp.vv<-tp.vv[1:2]
ntp<-length(tp.vv);
getci<-FALSE



if (ntp==1) {
  cc0<-c("模型 I","  一条直线效应");
  cc0<-c(cc0,"模型 II","  折点(K)","   < K 段效应 1","   > K 段效应 2","   2与1的效应差")
  if (is.na(tp.vv[1]) & chk==1 & clogit==0) getci<-TRUE;
} else {
```

```
  cc0<-c("模型 I","  一条直线效应");
  cc0<-c(cc0,"模型 II","  折点(K1,K2)","   < K1 段效应 1","   K1-K2 段效应 2","   > K2 段效应 3")
  cc0<-c(cc0,"   1与2的效应差","   3与2的效应差")
}
cc0<-c(cc0,"对数似然比检验");
if (getci) cc0<-c(cc0,"折点的95可信区间");
if (clogit==1 & chk==1) w<-c(w,"</br>Bootstrap for 95% CI of turning point was ignored for conditional logistic regression")
sink(paste(ofname,".lst",sep=""))
nn<-c("Outcome","Exposure",blvb);
if (prn=="Y") {
  for (j in 1:nx) {
    tt<-cc0;
    for (i in 1:ny) {
      if (is.na(tp.vv[1])) {tp.v<-get.tpval(i,j,NA,NA);} else {tp.v<-tp.vv;}
      if (ntp==1) tmpij<-get2lines(i,j,NA,tp.v);
      if (ntp==2) tmpij<-get3lines(i,j,NA,tp.v);
      if (getci) {tt<-cbind(tt,c(tmpij[[1]],getci4tp(i,j,NA,tp.v)));} else {tt<-cbind(tt,tmpij[[1]]);}
      nn<-rbind(nn,c(yb[i],xb[j],tmpij[[2]]))
    }
    tt<-rbind(c("Outcome: ",yb),tt)
    w<-c(w,paste("</br>For exposure:",xb[j]))
    w<-c(w,"</br><table border=3>", mat2htmltable(tt), "</table>")
  }
}
if (prn=="X") {
  for (i in 1:ny) {
    tt<-cc0;
    for (j in 1:nx) {
      if (is.na(tp.vv[1])) {tp.v<-get.tpval(i,j,NA,NA);} else {tp.v<-tp.vv;}
      if (ntp==1) tmpij<-get2lines(i,j,NA,tp.v);
      if (ntp==2) tmpij<-get3lines(i,j,NA,tp.v);
      if (getci) {tt<-cbind(tt,c(tmpij[[1]],getci4tp(i,j,NA,tp.v)));} else {tt<-cbind(tt,tmpij[[1]]);}
      nn<-rbind(nn,c(yb[i],xb[j],tmpij[[2]]))
```

```
    }
    tt<-rbind(c("Exposure: ",xb),tt)
    w<-c(w,paste("</br>For outcome:",yb[i]))
    w<-c(w,"</br><table border=3>", mat2htmltable(tt), "</table>")
  }
}
if (prn=="S") {
  for (i in 1:ny) {
    tt<-cc0;
    for (j in 1:nx) {
      nnij<-c(yb[i],xb[j])
      for (g in 1:nblv) {
        if (is.na(tp.vv[1])) {tp.v<-get.tpval(i,j,g,NA);} else {tp.v<-tp.vv;}
        if (ntp==1) tmpij<-get2lines(i,j,g,tp.v);
        if (ntp==2) tmpij<-get3lines(i,j,g,tp.v);
        if (getci) {tt<-cbind(tt,c(tmpij[[1]],getci4tp(i,j,g,tp.v)));} else {tt<-cbind(tt,tmpij[[1]]);}
        nnij<-c(nnij,tmpij[[2]])
      }
      nn<-rbind(nn,nnij)
    }
    tt<-rbind(c(blbl,blvb_),tt)
    w<-c(w,paste("</br>For outcome:",yb[i]))
    w<-c(w,paste("</br>For Exposure:",xb[j]))
    w<-c(w,"</br><table border=3>", mat2htmltable(tt), "</table>")
  }
}
sink()
w<-c(w,"</br>表中数据：HR (95% CI) Pvalue *P<0.05 **P<0.01 ***P<0.001")
w<-c(w,paste(c("</br>结果变量:",paste(yb,collapse="; ")),collapse=" "))
w<-c(w,paste(c("</br>暴露变量:",paste(xb,collapse="; ")),collapse=" "))
if (length(avname)==0) avb1<-"None";
w<-c(w,paste(c("</br>调整变量:",paste(avb1,collapse="; ")),collapse=" "))
if (smoothav>0) w<-c(w,". Restricted cubic spline smoothing were applied")
if (clogit==0) {
```

```
  tmpv<-c(vname.start,timevar); tmpv<-tmpv[!is.na(tmpv)];
  w<-c(w,paste(c("</br>Cox model 时间变量:", tmpv)))
}
```
